# Mutational profiling of mitochondrial DNA reveals an epithelial ovarian cancer‐specific evolutionary pattern contributing to high oxidative metabolism

**DOI:** 10.1002/ctm2.1523

**Published:** 2024-01-09

**Authors:** Fanfan Xie, Wenjie Guo, Xingguo Wang, Kaixiang Zhou, Shanshan Guo, Yang Liu, Tianlei Sun, Shengjing Li, Zhiyang Xu, Qing Yuan, Huanqin Zhang, Xiwen Gu, Jinliang Xing, Shujuan Liu

**Affiliations:** ^1^ Department of Obstetrics and Gynecology Xijing Hospital Fourth Military Medical University Xi'an China; ^2^ State Key Laboratory of Holistic Integrative Management of Gastrointestinal Cancers, Department of Physiology and Pathophysiology Fourth Military Medical University Xi'an China; ^3^ Institute of Medical Research Northwestern Polytechnical University Xi'an China; ^4^ State Key Laboratory of Holistic Integrative Management of Gastrointestinal Cancers, Department of Pathology Xijing Hospital and School of Basic Medicine Fourth Military Medical University Xi'an China

**Keywords:** epithelial ovarian cancer, evolutionary selection, metabolic remodelling, mitochondrial DNA, somatic mutations

## Abstract

**Background:**

Epithelial ovarian cancer (EOC) heavily relies on oxidative phosphorylation (OXPHOS) and exhibits distinct mitochondrial metabolic reprogramming. Up to now, the evolutionary pattern of somatic mitochondrial DNA (mtDNA) mutations in EOC tissues and their potential roles in metabolic remodelling have not been systematically elucidated.

**Methods:**

Based on a large somatic mtDNA mutation dataset from private and public EOC cohorts (239 and 118 patients, respectively), we most comprehensively characterised the EOC‐specific evolutionary pattern of mtDNA mutations and investigated its biological implication.

**Results:**

Mutational profiling revealed that the mitochondrial genome of EOC tissues was highly unstable compared with non‐cancerous ovary tissues. Furthermore, our data indicated the delayed heteroplasmy accumulation of mtDNA control region (mtCTR) mutations and near‐complete absence of mtCTR non‐hypervariable segment (non‐HVS) mutations in EOC tissues, which is consistent with stringent negative selection against mtCTR mutation. Additionally, we observed a bidirectional and region‐specific evolutionary pattern of mtDNA coding region mutations, manifested as significant negative selection against mutations in complex V (*ATP6*/*ATP*8) and tRNA loop regions, and potential positive selection on mutations in complex III (*MT‐CYB*). Meanwhile, EOC tissues showed higher mitochondrial biogenesis compared with non‐cancerous ovary tissues. Further analysis revealed the significant association between mtDNA mutations and both mitochondrial biogenesis and overall survival of EOC patients.

**Conclusions:**

Our study presents a comprehensive delineation of EOC‐specific evolutionary patterns of mtDNA mutations that aligned well with the specific mitochondrial metabolic remodelling, conferring novel insights into the functional roles of mtDNA mutations in EOC tumourigenesis and progression.

## Graphical abstract

This study provides a comprehensive delineation of EOC‐specific evolutionary patterns of mtDNA mutations based on large‐scale sample sequencing of the mitochondrial genome, which aligned well with specific mitochondrial metabolic remodelling. The mtDNA mutations were significantly associated with both mitochondrial biogenesis and prognosis of EOC patients, conferring novel insights into the functional roles of mtDNA mutations in EOC tumourigenesis and progression.

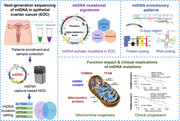

## 1 INTRODUCTION

Metabolic reprogramming is a prominent feature of malignant cancer, displaying significant heterogeneity among various tumour types.^1^ The prevailing perspective suggests that tumour cells enhance their growth and invasion by promoting aerobic glycolysis while impairing oxidative phosphorylation (OXPHOS).^2^ However, recent investigations indicate that certain cancers, such as colorectal cancer (CRC) and ovarian cancer (OC), heavily rely on OXPHOS, even in the presence of active glycolysis.^3^, ^4^ For instance, numerous studies have observed a substantial upregulation of genes linked to both OXPHOS and aerobic glycolysis in OC cells.^5^, ^6^, ^7^, ^8^ Mechanistically, the metabolic reprogramming of mitochondria in cancer cells is implicated in multiple oncogenic alterations, including the activation of nuclear oncogenes (e.g., MYC) and the loss of tumour suppressor gene function (e.g., TP53 and PTEN).^9^, ^10^ Nevertheless, these studies primarily focused on the impact of the nuclear genome, neglecting the role of the mitochondrial genome (mitochondrial DNA; mtDNA).

The mtDNA is the only genetic material outside the nucleus and contains two major regions, the control region (mtDNA control region; mtCTR) and the coding region (mtDNA coding region; mtCDR). The coding region encodes 13 mitochondrial proteins that play a vital role in the enzymatic complexes involved in OXPHOS, along with 22 transfer RNA molecules (tRNAs) and 2 ribosomal RNA molecules (rRNAs) that are necessary for protein synthesis specifically in mitochondria.^11^, ^12^ The absence of protective histones and an inefficient DNA repair system makes mtDNA highly prone to mutations, which are frequently observed in cancer genomes.^13^ Extensive research has been conducted to explore the impact of mtDNA mutations on cancer development and metabolic reprogramming.^14^, ^15^ For instance, Schöpf et al.^16^ discovered that mtDNA mutations contribute to the remodelling of OXPHOS in high‐grade prostate cancer. Furthermore, the presence of mtDNA truncating mutations is linked to a transcriptional phenotype characterised by the up‐regulation of OXPHOS genes, regardless of cancer lineage.^17^

Because of the disturbance to mitochondrial function and its influence on tumour biology, there is often a continuous selective pressure on mtDNA mutations during the growth and advancement of tumours.^13^ Recent investigations spanning multiple cancer types have demonstrated the prevalence of somatic mtDNA mutations, which display characteristic mutation patterns exclusive to cancer, potentially indicating varying evolutionary selection forces.^14^, ^18^ In our latest research, we performed a thorough examination of mtCTR mutations in 20 different human tumour types, unveiling three distinct evolutionary patterns that are exclusive to specific cancer types.^19^

Epithelial ovarian cancer (EOC) is the most prevalent form of OC and is strongly correlated with hormones. It is also the deadliest malignancy of the female reproductive system.^20^ Previous research has indicated that changes in mtDNA are functionally involved in the progression of EOC. For instance, Wang et al.^21^ discovered that alterations in mtDNA content are a significant genetic event in the development of ovarian carcinomas. Our recent pan‐cancer investigation also revealed distinct mutational patterns in the mtCTR between CRC and OC, despite both types of cancer relying heavily on OXPHOS.^19^ This suggests that mtDNA mutations have a cancer‐specific impact on tumour metabolism. Additionally, our recent study, which involved a large sample size, identified a special mode of mtDNA mutations specific to CRC, which may correspond to particular mitochondrial metabolic remodeling.^22^ However, the unique evolutionary patterns of somatic mtDNA mutations in EOC and their potential roles in metabolic remodelling have not been thoroughly examined.

In this study, we analysed a large dataset of somatic mtDNA mutations from both private and public EOC cohorts to characterise the evolutionary pattern specific to EOC. We also investigated the impact of these mutations on mitochondrial biogenesis and clinical progression, thus enhancing our comprehension of EOC tumourigenesis and metabolic remodelling.

## 2 MATERIALS AND METHODS

### 2.1 Patient enrolment and sample collection

Between January 2013 and February 2021, a total of 239 patients diagnosed with EOC and who underwent surgical resection were included in this study. Participants for this study were selected from Xijing Hospital, Fourth Military Medical University located in Xi'an, China. The specific inclusion requirements employed were as below: (1) histopathological confirmation of EOC; (2) no previous history of other types of cancer; (3) no prior treatment before the collection of samples. Based on the availability of different types of samples, the 239 EOC patients were divided into two groups: EOC cohort 1 (consisting of 89 patients) with fresh tumour tissues and cryopreserved preoperative blood samples from each patient, and EOC cohort 2 (consisting of 150 patients) with only formalin‐fixed and paraffin‐embedded (FFPE) tumour tissues and adjacent para‐tumour tissue samples. The relevant demographic and clinical characteristics of these two EOC cohorts are provided in Additional File 1: Table S1. Moreover, 56 patients with benign ovarian tumours (BOT) and 46 patients without any ovarian diseases were also included in this study, all recruited from Xijing Hospital. The study protocol was reviewed and approved by the Ethical Committee of Xijing Hospital under the reference number XJLL‐KY20212217. Written consent was obtained from each patient prior to their participation in the study. To ensure the inclusion of tumour tissues with at least 80% cancer cell content, the haematoxylin–eosin stained slides were carefully examined by two experienced pathologists, as previously described.^23^ Microdissection techniques were employed to acquire tissue samples with a greater proportion of tumour cells in instances where there was inadequate tumour cell content. As part of this study, fresh ovarian tissues and corresponding preoperative blood samples were collected from 56 patients with BOT and 46 patients without ovarian diseases.

### 2.2 DNA extraction, library preparation and capture‐based mtDNA sequencing

The previous description provided a detailed protocol for DNA extraction, library preparation and capture‐based mtDNA sequencing,^22^, ^24^ In this study, we introduce a modified method that utilises both mtDNA and reference nuclear DNA probes to construct a capture‐based NGS library. Initially, fresh tissue and peripheral blood mononuclear cell (PBMC) samples were subjected to genomic DNA extraction using the ENZA DNA Kit from Omega. For FFPE samples, the QIAamp DNA FFPE kit from QIAGEN was employed. Accurate quantification of DNA was accomplished using the Qubit 4.0 instrument from Thermo Fisher Scientific. To ensure optimal library construction, the genomic DNA was randomly fragmented, and fragments within the 300–500 bp range were selectively chosen. Subsequently, in‐house prepared biotinylated mtDNA probes were utilised to hybridise with the NGS libraries. The capture process specifically enriched mtDNA fragments of interest. Following the capture step, the mtDNA libraries were subjected to sequencing using the Illumina HiSeq XTen platform, generating paired‐end reads of 150 bp. This sequencing strategy allows for the comprehensive analysis of the captured mtDNA libraries.

### 2.3 MtDNA mutation calling and copy number calculation

The mtDNA mutation calling pipeline in this study was conducted following the methodology described in our previous study,^25^ with the present method validated to detect mutations at a heteroplasmy level of 1% or higher. In brief, the raw sequencing data were processed by fastp (v.0.20.1)^26^ to filter low‐quality reads and adaptor contaminations. The clean data were aligned to the revised Cambridge Reference Sequence (rCRS) and hgl9 reference using the BWA‐MEM algorithm (v.0.7.17).^27^ According to the GATK Best Practices Workflow, duplication reads and indel realignment were separately performed by Picard (v.l.81) and GATK IndelRealigner (v.3.2‐2). The SAMtools (v.l.7)^28^ software was employed to produce the pileup files, which facilitated the identification of mutations. In our study, we solely considered SNVs for further analysis. Furthermore, we examined mtDNA mutations in 24 fresh‐frozen tissue samples with heteroplasmy levels exceeding 1% using triple‐repeated capture‐based sequencing. Our results demonstrated a high level of consistency (Figure S1), thus confirming the dependability of our pipeline for the detection of low‐frequency mutations (VAF ≥ 1%). For each variant site, the heteroplasmy level (i.e., variant allele frequency; VAF) was determined by the percentage of mutant reads in the total reads. To accurately identify mtDNA mutations, the following filter conditions were employed: (1) a minimum of three reads supporting the alternative allele on each strand, (2) a sequencing coverage of at least 100× across the entire site, (3) VAF of no less than 1% on both strands and (4) exclusion of heterogeneity sites within repeat regions of the revised rCRS, specifically positions 66−71, 303−311, 514−523, 12418−12425 and 16184−16193. Moreover, stringent criteria were implemented to exclude C:G>A: T transversions where the VAF was ≤10%. This measure aimed to prevent artifacts resulting from oxidative 8‐oxoG during sample preparation.^29^ In addition, for FFPE DNA samples, a Strand Orientation Bias (SOB) score were calculated to estimate the probability of FFPE artifacts.^30^ Briefly, for mutations exhibiting low heteroplasmy levels (VAF ≤ 10%), only mutations with alternative allele supporting reads ≥20 and SOB scores ≤0.25 were retained. Somatic mtDNA mutations were identified as variants with VAF ≥ 1% in tumour tissues and wild‐type allele (VAF < 0.1%) in paired PBMC or adjacent non‐tumour tissues. A comprehensive summary of quality control data can be found in Additional File 1: Table S2. All samples were checked for cross‐contamination by mitoDataclean,^31^ and no cross‐contamination was observed. The information on mtDNA haplogroup status of each patient, the composition of haplogroups as well as the number of mutations in each haplogroup among different cohorts was summarised in Additional File 2. For a detailed overview of the original sequencing data of somatic mtDNA mutations (VAF > 0.8, *n* = 16) in private EOC cohort 1 was summarised in Additional File 3.

The calculation of mtDNA copy number in patient samples from exclusive cohorts was determined using the formula detailed in a previous study,^32^ $\mathrm{mtCN}\;=\frac{\mathrm{average}\;\mathrm{depth}\;\mathrm{of}\;\mathrm{mtDNA}}{\mathrm{average}\;\mathrm{depth}\;\mathrm{of}\;\mathrm{gDNA}}\;\times 2$, where mtCN is the mtDNA copy number, average depth of mtDNA is the mean coverage depths of whole mtDNA genome, average depth of gDNA is the mean coverage depths of six nuclear genome (nDNA) locations, which is captured by the six nDNA probes. To correct the reference bias, per‐individual consensus sequence of chrM instead of rCRS was used as reference genomes when estimating the mtDNA‐copy number. Public mtDNA copy number data were collected from the TCMA (http://bioinformatics.mdanderson.org/main/TCMA:Overview).^14^

### 2.4 Public mtDNA mutation datasets

Our previous publications have provided somatic mtDNA mutation data from 110 patients with hepatocellular carcinoma (HCC) (private HCC cohort) and 432 patients with CRC (public CRC cohort). These datasets were compared to explore the mutational patterns of mtDNA among different types of cancer.^22^, ^33^ To further validate the findings of the private mtDNA mutation datasets, we acquired a public mtDNA mutation dataset from whole‐genome sequencing (WGS) of tumour tissues from 118 EOC, 344 HCC and 62 CRC patients. The public dataset was obtained from the TCMA database (http://bioinformatics.mdanderson.org/main/TCMA:Overview).^14^ We conducted a comparative analysis of the same type of variants (specifically SNVs) in both private and public data, using a consistent VAF threshold of 1%. For more detailed information, please refer to Additional File 1: Table S3.

### 2.5 Annotation and functional impact prediction of somatic mtDNA mutations

All mtDNA mutations were subsequently annotated by ANNOVAR software.^34^ APOGEE2 scores were used to predict the functional impact of mitochondrial missense variants.^35^ Additionally, mutations in mtDNA tRNA encoding regions were categorised as benign or deleterious as described by Sonney et al.^36^ The annotation information of APOGEE2 scores for mitochondrial missense variants and Mitotip scores for mt‐tRNA mutations were summarised in Additional File 4.

### 2.6 Gene expression analysis

The mRNA expression level of *TFAM* and *TOMM20*, two nuclear‐encoded genes closely associated with mitochondrial biogenesis across multiple cancer types were analysed with a web server of GEPIA (http://gepia.cancer‐pku.cn/).^37^ In addition, we obtained RNA‐seq count data from the Cancer Genome Atlas (TCGA) and Genotype‐Tissue Expression Project (GTEx) database for 427 tissue samples with OC and 88 normal ovary (NOR) tissue samples. These datasets were utilised to investigate the mRNA expression levels of the TFAM and TOMM20 genes. IHC staining was performed on OC (*n* = 89) and NOR (*n* = 46) samples to assess the protein expression of TFAM and TOMM20, following the previously described methods.^38^ The qRT‐PCR analyses were carried out using the following primers: TFAM: forward, 5′‐AGCTCAGAACCCAGATGCAA‐3′, reverse, 5′‐CCACTCCGCCCTATAAGCAT‐3′; TOMM2: forward, 5′‐AGCTGGGCTTTCCAAGTTAC‐3′, reverse, 5′‐GTCAGATGGTCTACGCCCTT‐3′. Furthermore, we validated the differential expression of TFAM and TOMM20 using a high‐throughput mass spectrometry database that included 100 OC samples and 25 NOR tissue samples from The University of Alabama at Birmingham Cancer Data Analysis Portal (UALCAN) (http://ualcan.path.uab.edu/).^39^

### 2.7 Prognosis analysis

For the survival analysis, a group of 118 patients with mtDNA mutation data from the public cohort were included.^14^ To compare the survival, the utilisation of Kaplan–Meier curves and log‐rank tests (Mantel–Cox) was performed.

### 2.8 Statistical analysis

Statistical analyses were performed using GraphPad Prism version 8.3.0 software (San Diego, CA, USA). Prior to the analysis, G*Power software was utilised to calculate the sample power, which resulted in a statistical power of 90% at a significance level of .05. According to the power calculation, the analysis necessitated more than 100 patients diagnosed with EOC to estimate the sensitivity at a desired specificity of 0.95 or higher. In order to determine the mutation density, the number of mutations per sample was divided by the length of the region in kilobases (kb). To calculate the proportion of base substitution types within a specific region, the number of mutations with a specific substitution type was divided by the total number of mutations in that region. Before conducting any statistical analysis, normality of the data (e.g., heteroplasmic level, mtDNA copy number, mutation density) was tested using the Kolmogorov–Smirnov test. Additionally, the Levene test was employed to assess the homogeneity of variance among groups. Mean ± standard error of the mean (SEM) was used to express continuous variables, and the Mann–Whitney *U* test, Student's *t* test or one‐way ANOVA with Bonferroni's post hoc test was applied for analysis as needed. Categorical variables were assessed using the Chi‐square test. To examine the associations between measured variables, Spearman's rank correlation analysis was utilised. The Kolmogorov–Smirnov test was used to determine whether there were differential distributions between two groups of data. All reported *p* values were two‐tailed and considered statistically significant at a level of .05.

## 3 RESULTS

### 3.1 EOC exhibited highly unstable mitochondrial genome

In order to obtain a comprehensive profile of somatic mtDNA mutations in EOC tissues, we conducted capture‐based mtDNA deep sequencing on EOC, BOT and NOR tissues. In cohort 1, fresh EOC tissues and paired PBMCs were collected from 89 patients, while in cohort 2, FFPE EOC tissues and paired FFPE para‐EOC ovary tissues were collected from 150 patients. To assess the impact of different controls on somatic mutation detection, we analysed sequencing data from 15 EOC patients who had triple‐paired tumour, para‐tumour and PBMC samples. As shown in Figure S2, no obvious difference of somatic mtDNA mutations identified in EOC tissues was observed between two different controls. Due to similar mutational characteristics of the mtDNA sequencing data in both cohort 1 and cohort 2 (Figure S3), the two cohorts were finally combined into one private EOC cohort. Additionally, our private cohort included 56 patients with BOT and 46 patients without ovary diseases. We have summarised the sequencing data of mtDNA in Additional File 1: Table S2, with an average depth of 5599 ± 3069× in private EOC cohort 1, 4912 ± 3157× in private EOC cohort 2, 4013 ± 3054× in patients with BOT and 3326 ± 2789× in patients without ovary diseases. The median coverage of three private cohorts (EOC, BOT and NOR) for the entire mtDNA and mtCTR region was depicted in Figure S4.

**FIGURE 1:**
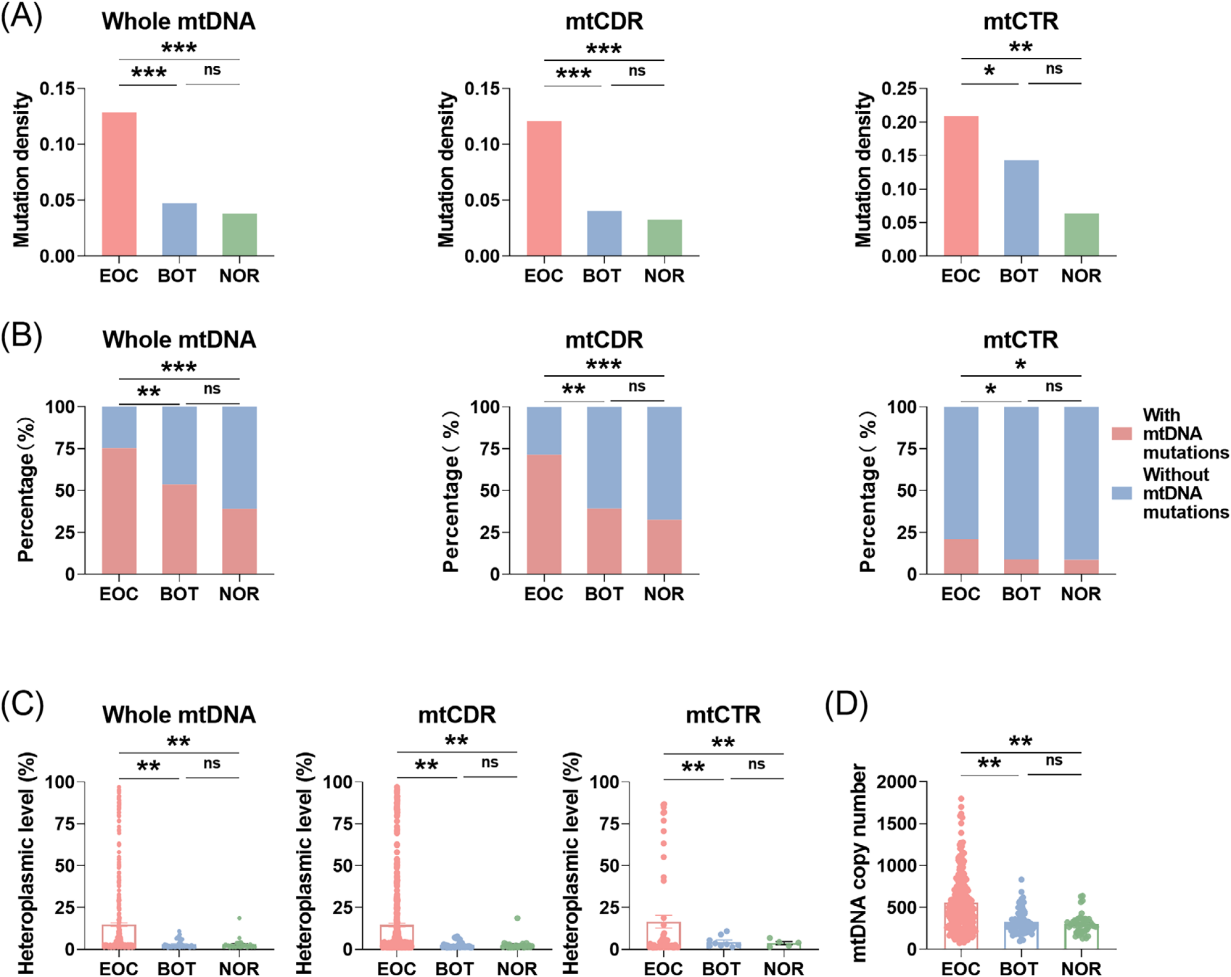
EOC exhibited highly unstable mitochondrial genome. (A) The percentage of tissues with somatic mutations in mtDNA, mtCDR and mtCTR among three tissue types. (B and C) The mutation density and heteroplasmic level of somatic mutations in mtDNA, mtCDR and mtCTR among three tissue types. Mutation density was calculated as the average number of mutations per sample per kilobase (kb). (D) The mtDNA copy number among three tissue types. EOC, epithelial ovarian cancer; BOT, benign ovarian tumour; NOR, normal ovary; mtCTR, mtDNA control region; mtCDR, mtDNA coding region. Data were expressed as mean ± SEM. Chi‐square test was used for data analysis in (A). One‐way ANOVA with Bonferroni's post hoc test was used for data analysis in panels (B–D). *p < .05; **p < .01; ***p < .001.

To investigate the potential functional roles of in the development of EOC tumours, we initially compared the mtDNA mutations and copy number (two major indicators of instability in the mitochondrial genome) in EOC, BOT and NOR tissues. As depicted in Circos plots (Figure S5), a total of 497 somatic mtDNA mutations were detected in EOC tissues, but only 44 in BOT tissues and 27 in NOR tissues, respectively. (More detailed information can be found in Additional File 4.) Further analysis indicated a significantly higher proportion of EOC tissue samples with somatic mutations in mtDNA, mtCDR and mtCTR when compared with both BOT and NOR tissues, with over 75% of EOC tissues containing at least one mtDNA mutation (Figure 1A). Notably, EOC tissues displayed increased mutability in mtDNA, mtCDR and mtCTR, as evidenced by higher mutation density and heteroplasmy levels compared with BOT and NOR tissues (Figures 1B and C). Similar results were obtained when focusing specifically on somatic mutations with a VAF greater than 5% (Figure S6). Moreover, EOC tissues exhibited significantly elevated mtDNA content relative to BOT and NOR tissues (Figure 1D). In addition, there was no significant difference in age among these study cohorts, indicating that age does not affect the distinct mutational characteristics observed. Overall, these findings highlight the high instability of mitochondrial genomes in EOC cells and their successive alterations during the progression of malignancy.

### 3.2 Mutations in mtCTR were subjected to more stringent negative selection in EOC

**FIGURE 2:**
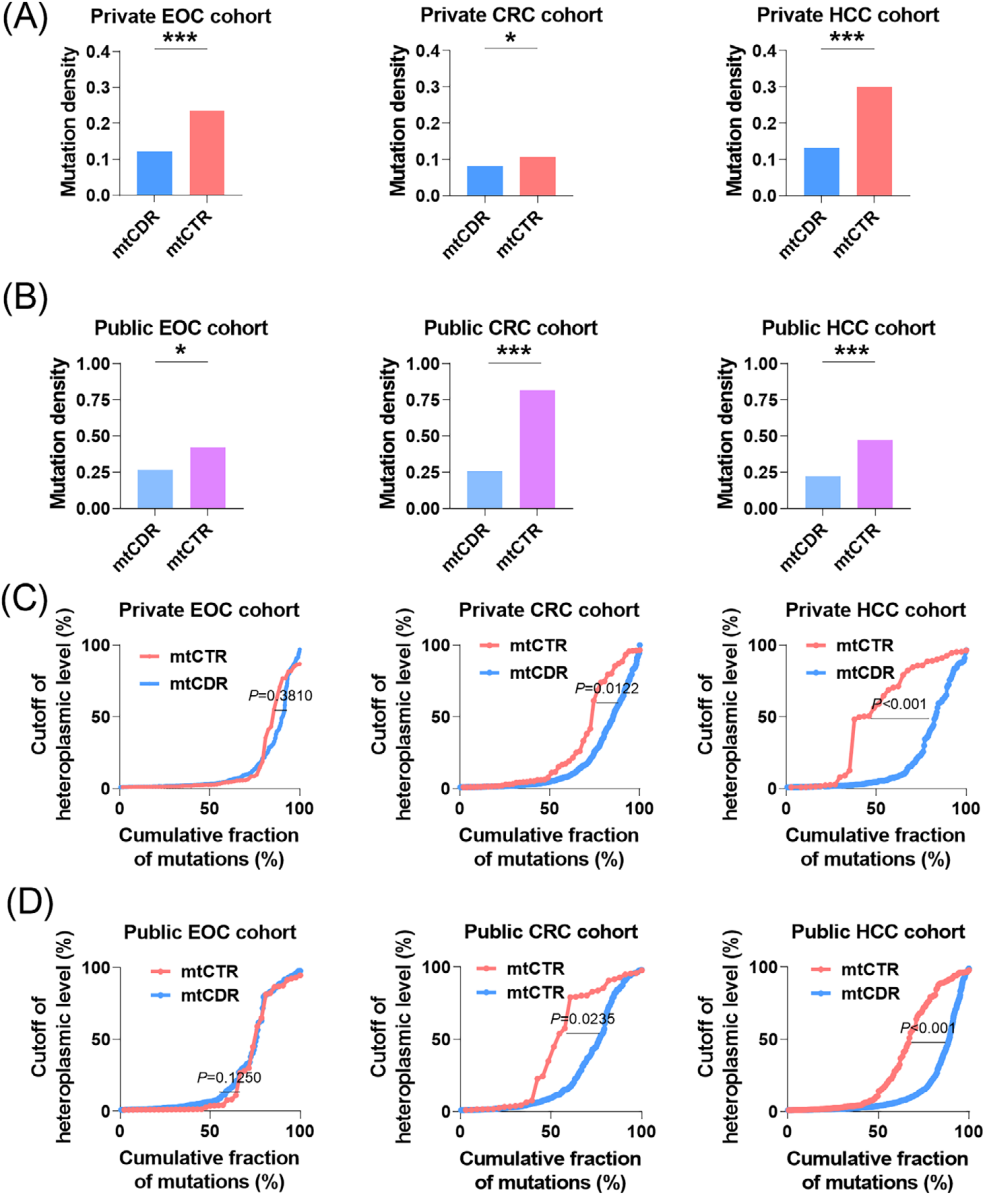
EOC displayed cancer‐specific evolutionary pattern of mtDNA mutations. (A and B) Mutation density for somatic mutations in mtCDR and mtCTR in private and public cohorts. (C and D) Cumulative distributions of mtCDR and mtCTR mutations in private and public cohorts. EOC, epithelial ovarian cancer; CRC, colorectal cancer; HCC, hepatocellular cancer; mtCDR, mtDNA coding region; mtCTR, mtDNA control region. Chi‐square test was used for data analysis in panels (A and B). Kolmogorov–Smirnov test was used for data analysis in panels (C and D). *p < .05; **p < .01; ***p < .001.

In order to investigate the cancer‐specific evolutionary patterns of mtDNA somatic mutations in EOC, a comparison was made between mtCTR and mtCDR mutations in three different types of cancer. As in HCC and CRC, the mtDNA mutations in EOC showed apparent strand bias, with L‐strand C>T mutations much more prevalent in mtCTR and L‐strand G>A mutations in mtCDR (Figure S7), indicating different evolutionary modes between mtCTR and mtCDR. Consistent with previous reports,^19^, ^22^ the mtCTR displayed a significantly higher mutation density in all three cancer types from both private and public cohort when compared with the mtCDR (Figures 2A and B). Notably, based on the cumulative distributions of heteroplasmy, cumulative fraction of mutations between the mtCTR and mtCDR were comparable in EOC from both private and public cohort, as opposed to HCC and CRC, where the mtCTR exhibited a much faster accumulation of high heteroplasmic mutations than the mtCDR (Figures 2C and D). These findings suggest that mtCTR mutations in EOC tissues undergo more rigorous negative selection compared with those in CRC and HCC tissues.

**FIGURE 3:**
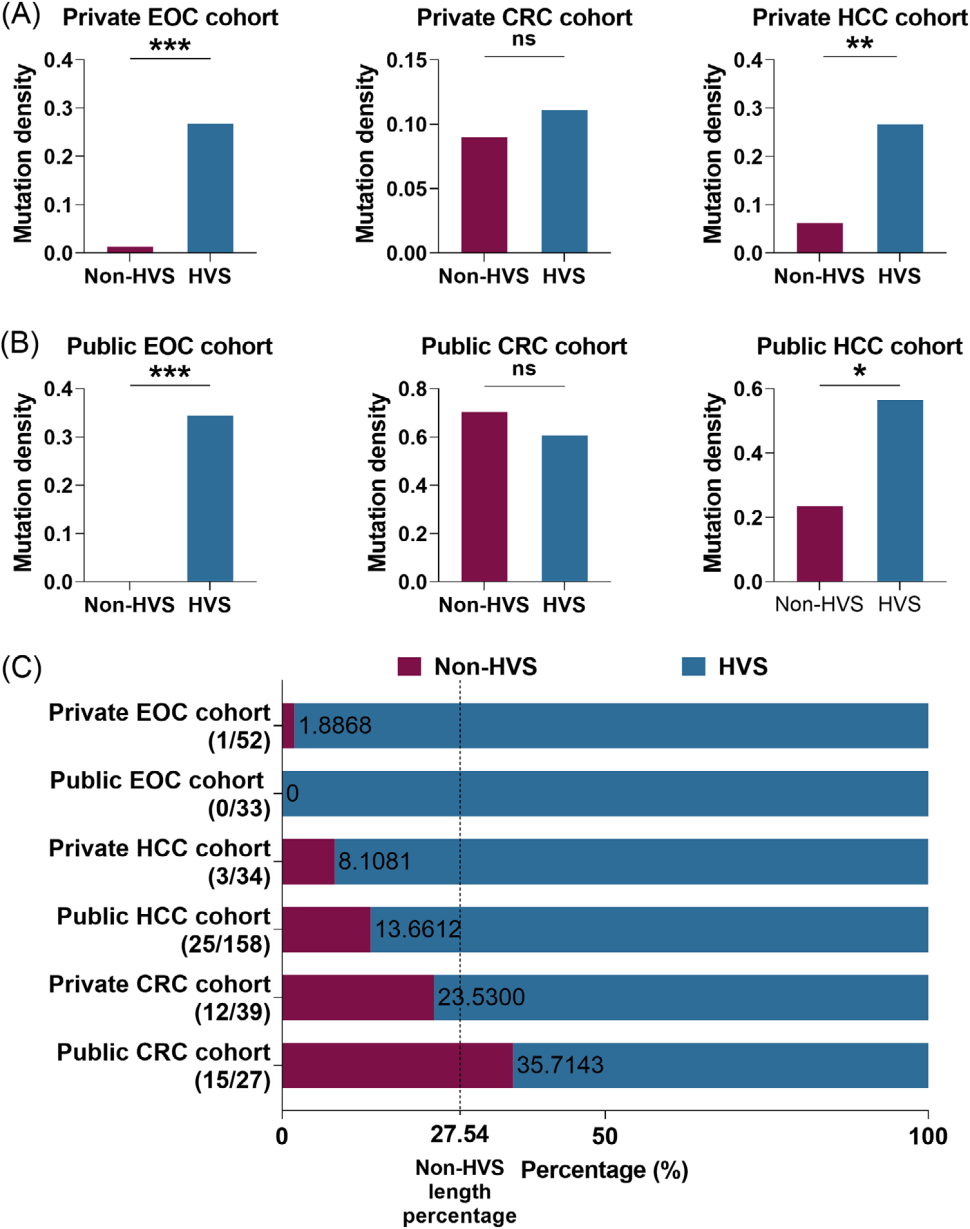
Mutations in mtCTR non‐HVS region were subjected to much strict negative selection in EOC. (A and B) Mutation density of mtCTR non‐HVS and HVS regions in private and public cohorts. © The proportion of somatic mutations in mtCTR non‐HVS and HVS regions for private and public cohorts. The value 27.54 of vertical dashed line indicated the length percentage of non‐HVS segment in mtCTR, which was used to represent possible percentage of non‐HVS mutations in mtCTR mutations when evolutionary selection is neutral. Mutation number in non‐HVS and HVS regions was shown in brackets. EOC, epithelial ovarian cancer; CRC, colorectal cancer; HCC, hepatocellular cancer; HVS, hypervariable region; mtCTR, mtDNA control region. Chi‐square test was used for data analysis in panels (A and B). *p < .05; **p < .01; ***p < .001.

The investigation of the specific mutational patterns of mtCTR in EOC was further conducted by comparing the density and proportions of mutations between the non‐HVS (309 bp) and HVS (813 bp) regions in different types of tumours. In contrast to CRC and HCC, which exhibited varying levels of mutation distribution in the non‐HVS region of mtCTR, EOC showed almost no mutations (1.89%, one in 53) in this region (Figure 3A). These observations were further validated using data from TCMA (Figure 3B). Consistently, the proportion of non‐HVS mutations within mtCTR displayed a weaker tendency compared with its length percentage in both EOC cohorts, in contrast to the higher enrichment of non‐HVS mutations in CRC and hepatocellular cancer (Figure 3C). On the other hand, no significant difference of mutation density was observed between the non‐HVS and HVS regions of mtDNA in both BOT and NOR samples (Figure S8). These results strongly indicate that OC imposes a stricter negative selection against non‐HVS mutations in mtCTR. Altogether, these findings suggest that the mtDNA landscape in EOC tissues is shaped by cancer‐specific selection pressure.

### 3.3 Mutations in mtDNA protein‐coding region presented complex‐specific evolutionary patterns in EOC

**FIGURE 4:**
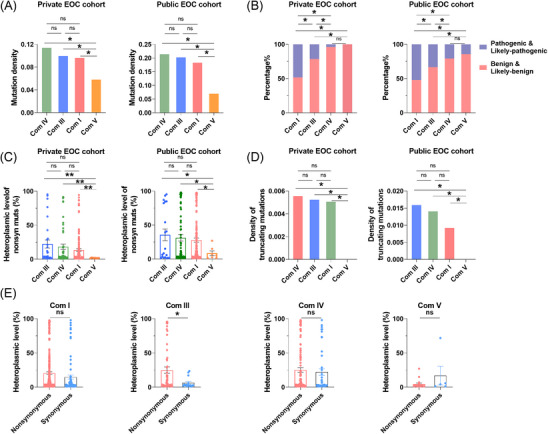
Mutations in mtDNA protein‐coding region presented complex‐specific evolutionary pattern in EOC. (A) Mutation density of mtDNA encoding‐mitochondrial respiration complexes (Com I, Com III, Com IV and Com V) in private and public EOC cohorts, presented in descending order, from the highest to the lowest. (B) The percentage of somatic mutations with different pathogenicity in mtDNA encoding‐mitochondrial complexes. (C) The heteroplasmic level of nonsynonymous somatic mutations in mtDNA encoding‐mitochondrial complexes. (D) Density of truncating mutations in mtDNA encoding‐mitochondrial respiration complexes. (E) The heteroplasmic level of nonsynonymous and synonymous somatic mutations in mtDNA encoding‐mitochondrial complexes. Mutation density of each complex was standardised based on the correlation of the mutation density of C_H_ > T_H_ with the DssH as previously described.^22^ Somatic mutations in mtDNA encoding‐mitochondrial complexes of private and public EOC cohorts were combined. EOC, epithelial ovarian cancer. Data were expressed as mean ± SEM. One‐way ANOVA with Bonferroni's post hoc test was used for data analysis in panels (A–C). The Mann–Whitney *U* test was used for data analysis in panel (E). *p < .05; **p < .01; ***p < .001.

We conducted a subsequent investigation into the evolutionary evidence of mutations in the mtDNA that encodes four mitochondrial complexes (complexes I, III, IV and V). The mutation density for each complex was standardised by comparing the mutation density of C_H_ > T_H_ with the DssH, as previously described.^22^ As depicted in Figure 4A, the standardised mutation density in Complex V exhibited a significantly lower value in comparison with the other three complexes, without any noticeable distinctions. Additionally, the pathogenicity analysis of variants revealed that complex V had significantly lower proportion of pathogenic and likely pathogenic mutations and lower heteroplasmy level of nonsynonymous mutations when compared with other three complexes (Figures 4B and C). Additionally, it is worth noting that unlike the other complexes, no truncating mutations were identified in complex V (Figure 4D). Similarly, there were no significant differences in the occurrence of truncating mutations among complexes I, III and IV. Furthermore, Furthermore, in contrast to mutations in the other complexes, complex III exhibited a significantly higher level of heteroplasmy in nonsynonymous mutations compared with synonymous mutations (Figure 4E). Our findings suggest that mtDNA mutations in complex V may be under restrict negative selection due to possible impairment of ATP synthesis and nonsynonymous mutations in complex III may confer a functional advantage in certain stages of EOC progression and thus be subject to possible complex‐specific positive selection.

### 3.4 Mutations in mtDNA tRNA were prone to region‐specific evolutionary selection in EOC

**FIGURE 5:**
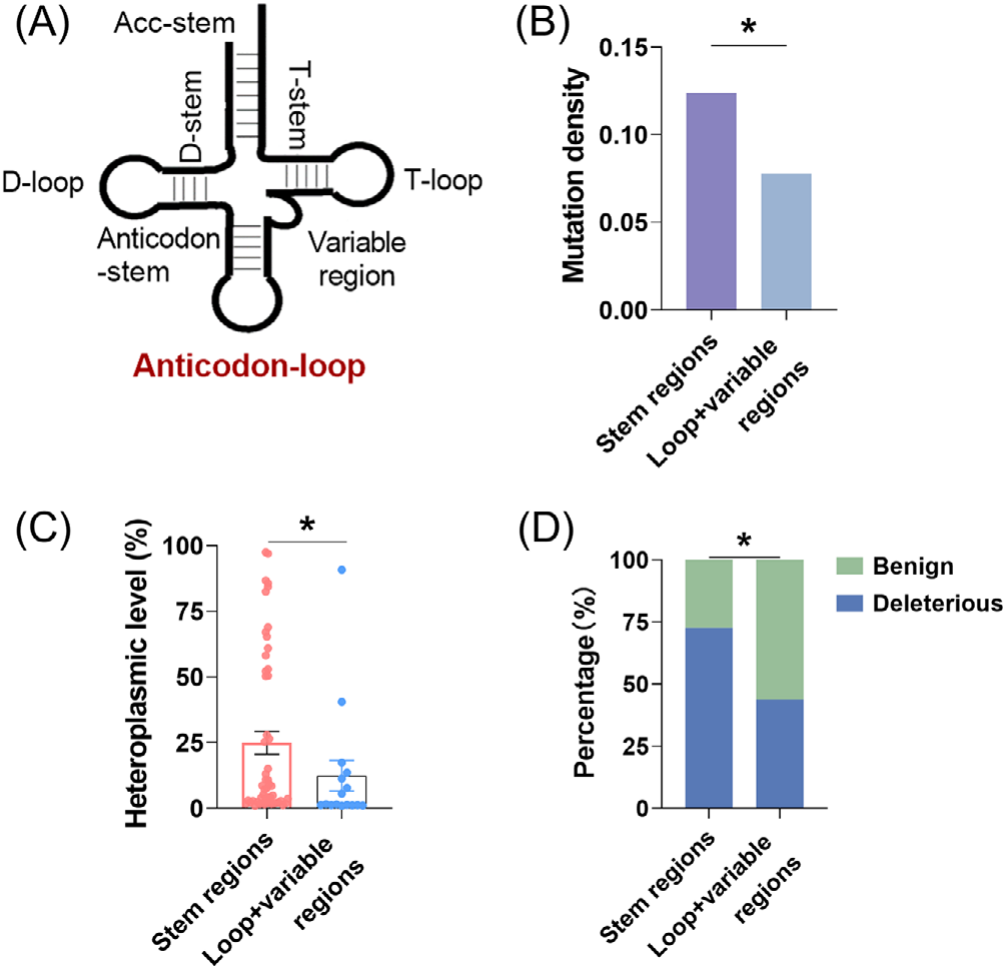
Mutations in mtDNA tRNA were prone to region‐specific evolutionary selection in EOC. (A) Diagram of tRNA functional units. (B and C) Mutation density and heteroplasmic level of somatic mutations in mitochondrial tRNA stem (*n* = 55) and non‐stem regions (*n* = 16, loop and variable region) in combined EOC cohort. (D) Percentage of benign and deleterious mutations in mitochondrial tRNA stem and non‐stem regions. Somatic mutations in mitochondrial tRNAs coding regions of private (*n* = 37) and public (*n* = 34) EOC cohorts were combined. Data were expressed as mean ± SEM. Chi‐square test was used for data analysis in (B) and (D). The Mann–Whitney *U* test was used for data analysis in panel (C). *p < .05; **p < .01; ***p < .001.

Furthermore, we investigated whether evolutionary selection also impacted somatic mutations present in mitochondrial tRNA genes within EOC tissues. Based on the secondary structures, tRNAs were categorised into two functional units: the stem regions and the loop/variable regions (as depicted in Figure 5A). Our findings demonstrate that both the density of mutations and the level of heteroplasmy in the tRNA loop/variable regions were notably lower compared with the stem regions (Figures 5B and C). Moreover, mutations in tRNA stem regions were annotated as significantly elevated pathogenicity when compared with that of those in loop/variable regions (Figure 5D). Meanwhile, further analysis revealed that almost no mutations (only G12300A, with a low heteroplasmy level of 1.39%) were detected in the tRNA anticodon loop region corresponding to the first or second nucleotide of the complementary codon (see details in the Additional File 4), indicating that mutations in this region have experienced strong negative selection because of their significant roles in the structure and function of tRNA molecules.^40^ Together, these findings indicate that EOC may preferentially deplete somatic tRNA mutations in a region‐specific manner.

### 3.5 EOC showed much active mitochondrial biogenesis and oxidative metabolism

**FIGURE 6:**
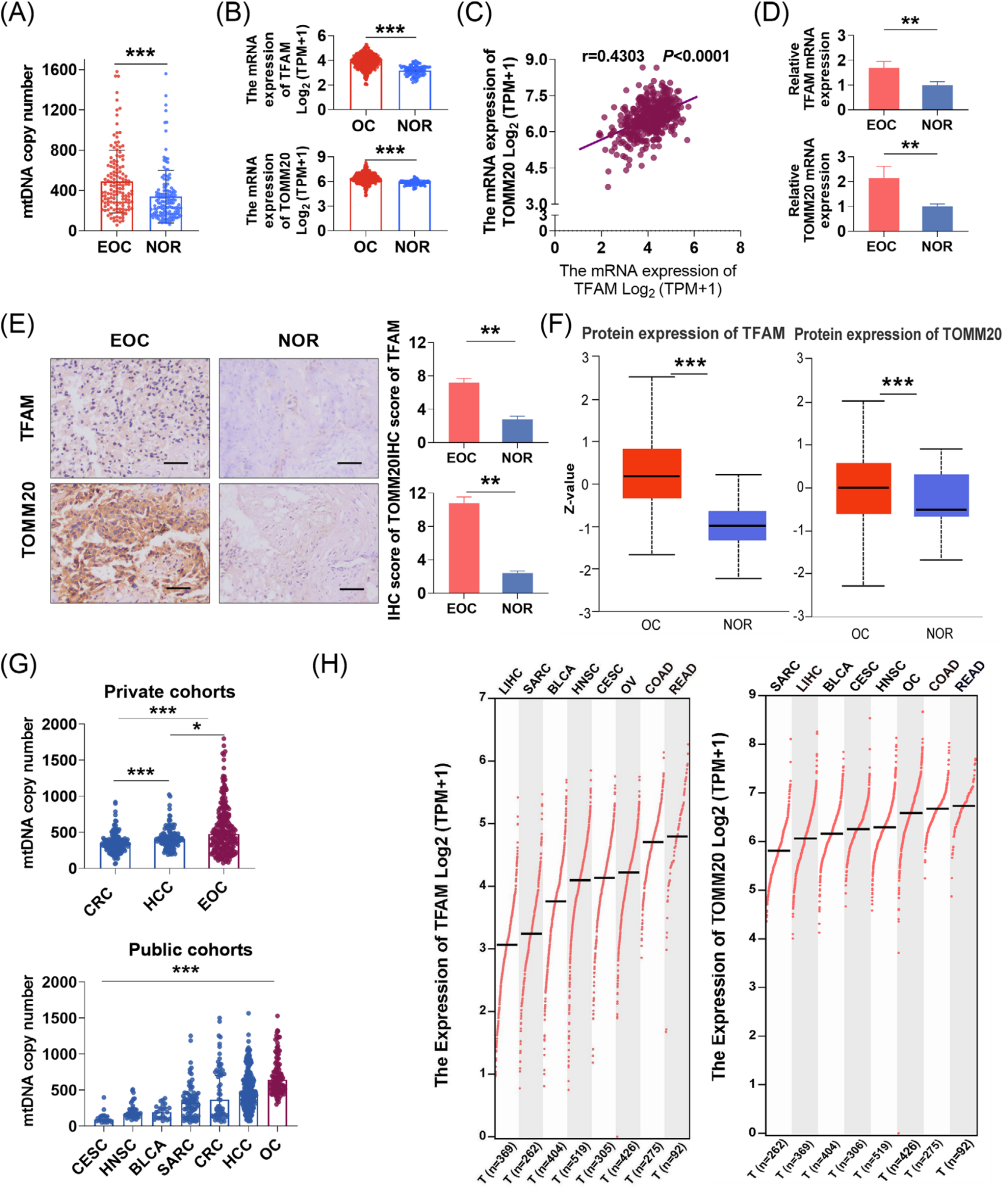
EOC showed much active mitochondrial biogenesis. (A) NGS‐based analysis of mtDNA copy number in paired EOC (*n* = 150) and NOR (*n* = 150) tissues in private EOC cohort 2. (B) Expression of TFAM and TOMM20 mRNA in OC (*n* = 427) and NOR (*n* = 88) tissues based on RNA‐seq counts retrieved from TCGA and GTEx database, respectively. (C) Spearman correlation analysis between the level of TFAM and TOMM20 mRNA expression in OC (*n* = 427) tissues based on RNA‐seq counts retrieved from TCGA. (D) qRT‐PCR analyses for TFAM and TOMM20 mRNA expression in EOC tissues (*n* = 89) from private EOC cohort 1 and NOR tissues (*n* = 46) from private cohort without ovary disease. (E) Representative immunohistochemistry (IHC) staining images and quantification of mitochondrial TFAM and TOMM20 in EOC tissues (*n* = 89) from private EOC cohort 1 and NOR tissues (*n* = 46) from private cohort without ovary disease. Scale bars: 25 μm. (F) Protein expression of TFAM and TOMM20 in OC (*n* = 100) and NOR (*n* = 25) tissues based on mass spectrometry data from the Clinical Proteomic Tumour Analysis Consortium (CPTAC). Protein expression values downloaded from the CPTAC data portal were log2 normalised in each sample. Then a *Z*‐value for each sample for each protein was calculated as standard deviations from the median across samples. (G) Distributions of mtDNA copy number by cancer tissue type in private and public cohorts. (H) Expression of TFAM and TOMM20 mRNA by cancer tissue type based on RNA‐seq counts retrieved from TCGA database. EOC, epithelial ovarian cancer; NOR, normal ovary; BLCA, bladder urothelial carcinoma; CESC, cervical squamous cell carcinoma and endocervical adenocarcinoma; COAD, Colon Cancer; CRC, colorectal cancer; HCC, hepatocellular cancer; HNSC, head and neck squamous cell carcinoma; READ, Rectal Cancer; SARC, Sarcoma. Data were expressed as mean ± SEM. The Mann–Whitney *U* test was used for data analysis in (A), (B), (D) and (F). The Student's *t*‐test was used for data analysis in panel (E). One‐way ANOVA with Bonferroni's post hoc test was used for data analysis in panel (G). *p < .05; **p < .01; ***p < .001.

To further understand the potential functional effects of somatic mtDNA mutations on the development of EOC, we conducted a systematic evaluation of mitochondrial biogenesis in EOC tissues. EOC tissues presented a significantly higher copy number of mtDNA compared with NOR tissues (Figure 6A). Additionally, RNA‐seq, qRT‐PCR, IHC and mass spectrometry data analyses indicated that OC tissue showed significantly higher expression of mitochondrial biomass markers at both protein and mRNA levels when compared with NOR tissue, including mitochondrial transcription factor A (TFAM, binding to mtDNA in nucleoids) and mitochondrial outer membrane translocase (TOMM20) (Figures 6B–F). We further analysed the level of mitochondrial biogenesis in different cancer types based on the mtDNA copy numbers and the expression levels of two mitochondrial biomass markers (Figures 6G and H). Our data showed extensive variation across cancer types, with EOC ranking top. These findings strongly suggest that OC tissues exhibited active mitochondrial biogenesis responsible for a high metabolic dependence on OXPHOS,^4^, ^5^ supporting a critical role for mitochondrial oxidative metabolism in the growth of OC.

### 3.6 MtDNA mutations in EOC was associated with mitochondrial biogenesis and clinical outcome

**FIGURE 7:**
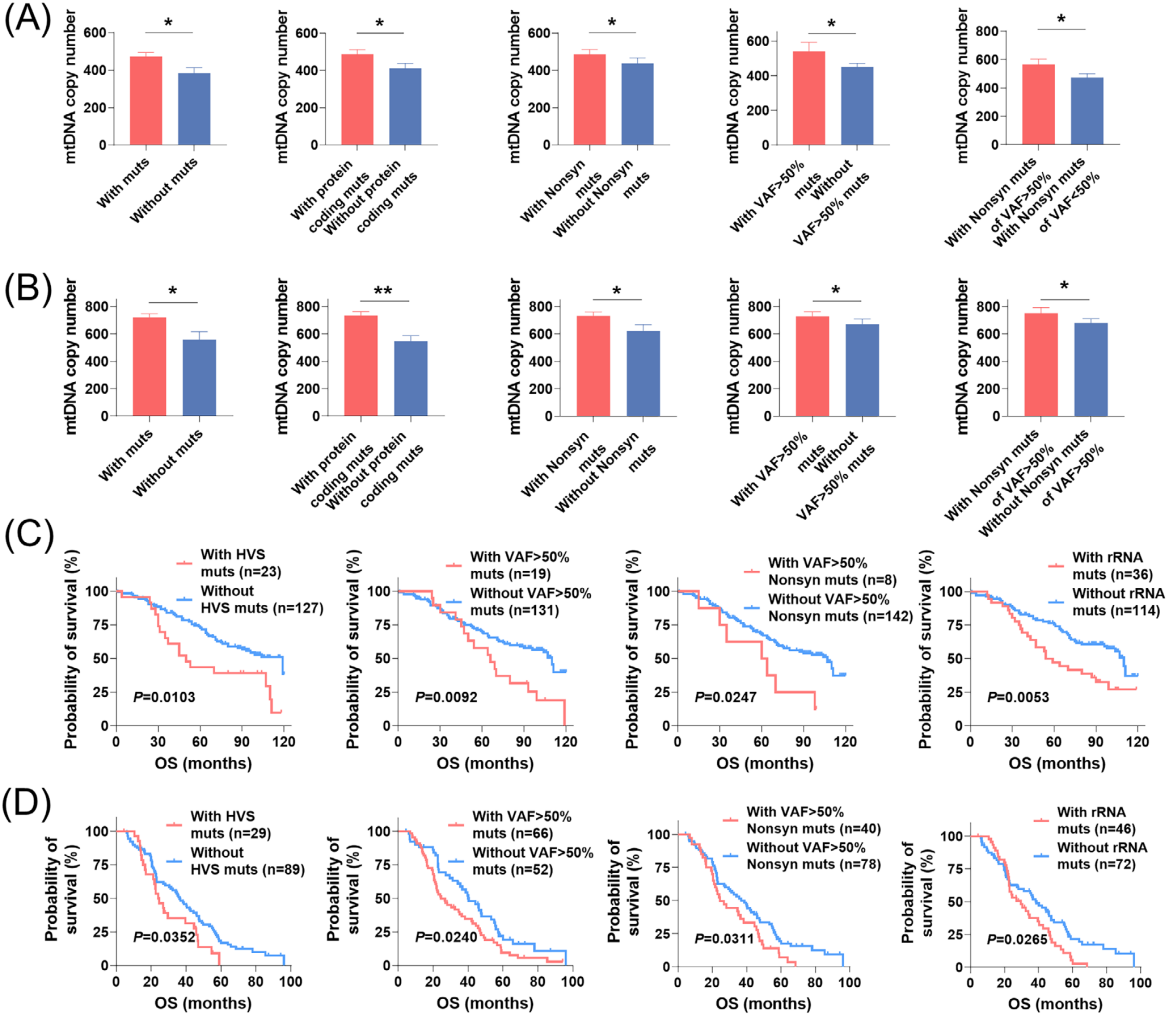
MtDNA mutations was notably associated with mitochondrial biogenesis and clinical outcome of EOC. (A and B) Comparison of mtDNA copy number between patients with and without mtDNA mutations, patients with and without protein‐coding mutations, patients with and without nonsynonymous mutations, patients with and without high VAF mutations, patients with and without high VAF nonsynonymous mutations, patients with and without mtCTR mutations, and patients with and without tRNA mutations in private (A) and public (B) EOC cohorts. (C and D) Kaplan–Meier curve analysis of overall survival (OS) between patients with and without HVS mutations, patients with and without high VAF mutations, patients with and without high VAF nonsynonymous mutations and patients with and without rRNA mutations in private (C) and public (D) EOC cohorts. Mutations with VAF higher than 50% were defined as high VAF mutations. VAF, variant allele frequency; Nonsyn muts, nonsynonymous mutations; mtCTR, mtDNA control region. Com I, mitochondrial complex I. The Mann–Whitney *U* test was used for data analysis in panel (A and B). *p < .05; **p < .01.

Considering the critical role of mtDNA in mitochondrial oxidative metabolism,^16^ we investigated the association between mtDNA mutations stratified by functional regions and mtDNA copy number in EOC (Figure 7A). Copy number of mtDNA was significantly higher in EOC tissues with mutations than in those without mutations. Further analysis revealed that mtDNA copy numbers were significantly increased in EOC tissues with potential functional mutations when compared with tissues without those mutations, which included protein‐coding mutations, nonsynonymous mutations, mutations with high heteroplasmy levels (VAF > 50%) and nonsynonymous mutations with VAF > 50%, suggesting that these mutations may play direct or indirect roles in mitochondrial biogenesis. Moreover, no significant difference in mtDNA copy number was found between EOC tissues with and without mtCTR mutations, and between EOC tissues with and without tRNA mutations (Figure S9A), which is highly consistent with negative evolutionary selection in both mtCTR and tRNA regions. The findings were recapitulated in OC (*n* = 118) tissues from the TCMA dataset (Figures 7B and S9B).

To gain deeper insight into the influence of mutation patterns on patient phenotype, we further evaluated whether mtDNA mutations is prognostically meaningful in OC based on private EOC dataset (Figures 7C and S9C). Our findings revealed that patients with HVS mutations had a worse prognosis compared with those without these mutations. Furthermore, mutations with a higher heteroplasmy level, especially nonsynonymous mutations were associated with a poorer prognosis, supporting the hypothesis that heteroplasmic dosage is likely a critical determinant of the phenotype produced by mtDNA mutations. Additionally, patients with rRNA mutations also showed poorer prognosis than those without rRNA mutations. However, no significant difference in overall survival was found between EOC patients with and without mtDNA mutations, and between EOC patients with and without mutations in mtCDR, and between EOC patients with and without tRNA mutations. Very similar findings were also observed in the public OC cohort (Figures 7D and S9D). Taken together, mtDNA mutations were associated with mitochondrial biogenesis and increased risk of EOC progression, suggesting that mtDNA mutations may be involved in the development of EOC and could be used as prognostic biomarkers.

## 4 DISCUSSION

In this study, we comprehensively characterised the specific evolutionary pattern of mtDNA somatic mutations in EOC. We accomplished this by analysing capture‐based mtDNA NGS sequencing data from both private and public EOC groups. Our study revealed several important discoveries. First, it was observed that the mitochondrial genome undergoes frequent disturbances in EOC, exhibiting significantly higher rates of point mutation and copy number alteration compared with non‐cancerous ovary tissue. Additionally, the evolutionary pattern of somatic mtDNA mutations in EOC displayed distinct characteristics exclusive to cancer. These characteristics entail a combination of strict selection for somatic mutations in the mtCTR, along with negative selection against mutations in mtDNA complex V (ATP6/ATP8) and tRNA loop areas. Moreover, there may be potential positive selection for mutations within the mtDNA complex III (MT‐CYB) gene. Last, the EOC‐specific evolutionary pattern of somatic mtDNA mutations was found to have a close association with both mitochondrial biogenesis and the prognosis of EOC patients.

An expanding body of research has evidenced that mtDNA mutations are frequently occurring genetic occurrences in all tumour types.^13^ Recently, Yuan et al.^14^ extensively characterised the molecular features of mtDNA mutations across 38 tumour types using WGS data from 2658 tissue samples. Their findings imply that mtDNA mutations may undergo distinct evolutionary selection in different cancers.^14^ Nevertheless, due to limited sample sizes and mutation numbers, a comprehensive investigation into cancer‐specific patterns of mtDNA mutation and evolutionary selection has been constrained. To address this limitation, we previously conducted large‐scale sequencing studies on the mitochondrial genomes of HCC and CRC, thoroughly mapping out the evolutionary patterns of mtDNA mutations unique to these cancer types.^22^, ^41^ Notably, HCC demonstrated a significant positive selection of mtCTR mutations, indicating a notably higher level of heteroplasmy in HCC compared with non‐HCC tumours. This association is closely linked to mitochondrial biogenesis and patient prognosis.^41^ On the contrary, a less stringent evolutionary selection of mtCTR mutations has been observed in CRC, resulting in no noteworthy impact on clinicopathological characteristics, thus suggesting a more ‘passenger’ role.^22^ Our ongoing research has revealed a unique evolutionary pattern of mtDNA mutations specific to EOC, which is exemplified by a combination of strict selection in mtCTR, mt‐complex V and mt‐tRNA loop regions, along with potential positive selection of mt‐complex III (MT‐CYB) mutations. Sanchez‐Contreras et al.^42^ have previously documented the tissue‐specific range of somatic mtDNA mutations in normal tissues, highlighting the liver and kidney tissues as having the highest frequency of mutations. Although somatic mtDNA mutations in normal tissues may be influenced by potential evolutionary selection in a tissue‐specific manner, these findings provide strong evidence that mtDNA mutations undergo cancer‐specific evolutionary selection, thereby supporting the functional involvement of somatic mtDNA mutations in mitochondrial metabolic remodelling and tumour progression.

The 1122 bp mtCTR, plays a critical role in the replication and transcription of mtDNA. It consists of two types of segments: the hypervariable segments (HVS 1−3), which are highly variable, and the non‐HVS segments, which show less variability. These segments contain essential regulatory elements necessary for proper mtDNA functioning.^43^ In a recent pan‐cancer study conducted by our team, we observed that mtDNA mutations in the mtCTR exhibit high variability among different tumour types. Three distinct evolutionary patterns were identified: relaxed, moderate and strict constraint types.^19^ In the present study, the stringent negative selection for mtCTR non‐HVS mutations was further validated in EOC using a much large sequencing dataset. Moreover, our study also revealed that the mtCTR HVS mutations in EOC was subjected to significant negative selection, as evidenced by the delayed heteroplasmic mtCTR mutation accumulation specifically in EOC. Together, these findings render the strict selection of mtCTR mutations as one most salient feature of the EOC‐specific pattern of selection for mtDNA mutation. Furthermore, our data indicated no notable effect of mtCTR mutations on mtDNA content in OC. Given the crucial role of mtCTR in mtDNA replication, we hypothesise that OC cells are intolerant of mtCTR mutations due to highly demanded mitochondrial biogenesis, which results in strict purifying selection against mutations in mtCTR, especially non‐HVS mutations. This highlights the importance and necessity of maintaining functional mtCTR and mitochondrial biogenesis in EOC. Interestingly, based on our recent pan‐cancer study, several hormone‐related tumours, including prostate cancer, thyroid cancer and OC, appear to share similar strict selection of mtCTR mutations,^19^ suggesting the possible role of hormone in shaping evolutionary pattern of mtDNA mutations, which warrants further exploration.

Our findings also indicate that mutations in the coding region of mtDNA in EOC are under complex and region‐specific selection. Specifically, there is a negative selection against mutations in mtDNA complex V (ATP6/ATP8) and the tRNA loop regions, particularly in the tRNA anticodon loop region. This suggests that the maintenance of mitochondrial function, particularly in mitochondrial protein translation and ATP generation, is crucial for EOC cells.  Hence, metabolic remodelling in EOC may not be geared towards the inhibition of mitochondrial respiration, supporting the significance of functional mitochondrial metabolism in OC.^4^, ^8^ Consistent with this view, single‐cell RNA sequencing data have revealed that genes associated with OXPHOS are significantly upregulated in OC cells.^5^ A remarkable increase of mtDNA copy number was also observed in EOC cells. As a result, the evident link between mutations in the coding region of mtDNA and the generation of mitochondria, as well as the prognosis of EOC, strongly implies that these mutations significantly impact the respiration of mitochondria and the clinical outcomes of patients with EOC.

Amounting studies have shown that OC cells are highly dependent on mitochondrial OXPHOS.^4^, ^7^, ^44^ Accordingly, targeting OXPHOS has been identified as an important strategy for the treatment of OC.^45^, ^46^, ^47^ However, the exact mechanism underlying remodelling of mitochondrial metabolism in OC cells has not been fully elucidated. In the present study, our data indicated that EOC patients carrying nonsynonymous mutations with a high heteroplasmy level (VAF > 50%) tended to have high mtDNA copy numbers and poor prognosis. Accordingly, Koc et al.^48^ have reported that the high demand for oxidative metabolism in OC cells induces the mitochondrial biogenesis. It has been reported that the absolute copy number of wild‐type mtDNA is an important determinant of the pathological impact of mtDNA variants.^13^  In a study conducted by Filograna et al.,^49^ it was observed that augmenting the copy quantity of mtDNA alleviates the pathological repercussions of a heteroplasmic mtDNA mutation in tRNA^Ala^ and resolves the COX deficiency observed across various tissues. Consistently, Larsson and coworkers^50^ have provided evidence demonstrating that an increased copy number of mtDNA effectively mitigates the infertility phenotype induced by mtDNA mutations in mice. According to our research, it is also indicated that certain mutations in EOC tissues' mtDNA may have significant impact, acting as ‘driver’ mutations. These mutations, specifically found in mtDNA complex III (MT‐CYB), might be subject to positive selection. The MT‐CYB gene is responsible for encoding the cytochrome b protein within complex III, which is involved in the process of OXPHOS. Studies have shown that mutations in the MT‐CYB gene can actually heighten the production of reactive oxygen species (ROS) within cancer cells.^51^ Additionally, *Klimova T et al*. highlighted the significance of ROS produced by mitochondrial complex III in the activation of hypoxia‐inducible factor. This finding contributes to our understanding of how cancer, specifically EOC, adapts metabolically to hypoxic conditions.^52^ Consistent with this, our recent study has also identified recurrent and heteroplasmy‐elevated mtDNA mutations from primary OC lesions to metastasis lesions, suggesting the presence of driver mutations conferring OC metastatic advantage.^23^ Similarly, Grandhi et al.^53^ has suggested that mtDNA mutations may be under positive selection in kidney and thyroid cancers. However, the specific role of mutations in *MT‐CYB* gene in EOC need further in‐depth study in the future. Our investigation revealed distinct patterns of somatic mutations in the control and coding regions of mitochondria, specifically associated with EOC. These findings carry significant implications for mitochondrial biogenesis and the prognosis of patients with EOC.

## 5 CONCLUSIONS

This study provides a comprehensive delineation of EOC‐specific evolutionary patterns of mtDNA mutations based on large‐scale sample sequencing of the mitochondrial genome, which aligned well with specific mitochondrial metabolic remodelling. This study confers novel insights into the functional roles of mtDNA mutations in EOC tumourigenesis and progression.

## AUTHOR CONTRIBUTION

F. X., W. G. and X. W. carried out the sample collection, performed the data analysis and drafted the manuscript. S. G. and Q. Y. collected the public data and participated in the bioinformatics analyses. Z. X., Y. L., T. S. and H. Z. performed the laboratory experiments. S. L. and X. W. performed the statistical analysis. J. X., K. Z. and X. G. participated in the design of the study and performed the draft revision. S. L. and J. X. conceived of the study, and participated in its design and coordination and helped to revise the manuscript. All authors read and approved the final manuscript.

## CONFLICT OF INTEREST STATEMENT

The authors declare that they have no competing interests.

## CONSENT FOR PUBLICATION

Not applicable.

## ETHICS STATEMET AND CONSENT TO PARTICIPATE

This study was approved by the Ethical Committee (No. XJLL‐KY20212217) of Xijing Hospital. Written informed consent was obtained by all participants.

## Supporting information

Supporting Information

Supporting Information

Supporting Information

Supporting Information

## Data Availability

Public somatic mtDNA mutation data are available within three publications^14^, ^22^, ^33^ and our private mtDNA mutation data are available within the Additional File 4. The sequencing data have been uploaded to the Genome Sequence Archive for Human (GSA‐Human) under accession PRJCA006830 (http://bigd.big.ac.cn/gsa‐human). RNA‐seq counts data were downloaded from the GTEx (https://www.gtexportal.org) and TCGA (https://www.cancer.gov/tcga). The raw sequencing data and the source code of our pipeline underlying this article are available upon request.
