# Supplementary material for: Mutational profiling of mitochondrial DNA reveals an epithelial ovarian cancer‐specific evolutionary pattern contributing to high oxidative metabolism
Source: Clin Transl Med. 2024 Jan 9;14(1):e1523. doi: 10.1002/ctm2.1523 (PMC10775184; doi:10.1002/ctm2.1523)
Supplement: Supplementary file 1 — Supporting Information [file CTM2-14-e1523-s001.docx]

**Mutational profiling of mitochondrial DNA reveals an epithelial ovarian cancer-specific evolutionary pattern contributing to high oxidative metabolism**

Fanfan Xie^1,2#^, Wenjie Guo^2#^, Xingguo Wang^1#^, Kaixiang Zhou^2^, Shanshan Guo^2^, Yang Liu^2^, Tianlei Sun^2^, Shengjing Li^2^, Zhiyang Xu^1^, Qing Yuan^4^, Huanqin Zhang^2^, Xiwen Gu^3^, Jinliang Xing^2*^, Shujuan Liu^1*^

**Supplementary materials include 9 figures and 3 tables:**

**Figure S1.** Venn diagram of mtDNA mutations with more than 1% heteroplasmy level in 24 fresh-frozen tissue samples by triple-repeated capture-based sequencing.

**Figure S2.** Venn diagram of mtDNA mutations with more than 1% heteroplasmy level in triple-paired tumor, para-tumor and PBMC samples from 15 EOC patients.

**Figure S3.** The characteristics of mtDNA mutations were comparable between private EOC cohorts 1 and 2.

**Figure S4.** Comparison of mtDNA mutations with VAF > 5% among different groups.

**Figure S5.** Circos plots showing the frequency and distribution of somatic mtDNA mutations from EOC, BOT, and NOR tissues in private cohort.

**Figure S6.** MtDNA mutations in EOC showed strong region and strand biases.

**Figure S7.** Mutation density of mtCTR non-HVS and HVS regions in private BOT and NOR cohorts.

**Figure S8.** The association between mtDNA mutations and mitochondrial biogenesis as well as clinical outcome of EOC.

**Figure S9**. Median coverage of whole mtDNA and mtCTR in private EOC, BOT and NOR cohorts.

**Table S1.** Clinical characteristics of two private epithelial ovarian cancer (EOC), one private BOT, and one private NOR cohorts.

**Table S2****.** Summary of mtDNA sequencing data from private cohorts.

**Table S3****.** Summary of mtDNA somatic mutations in public cohorts.


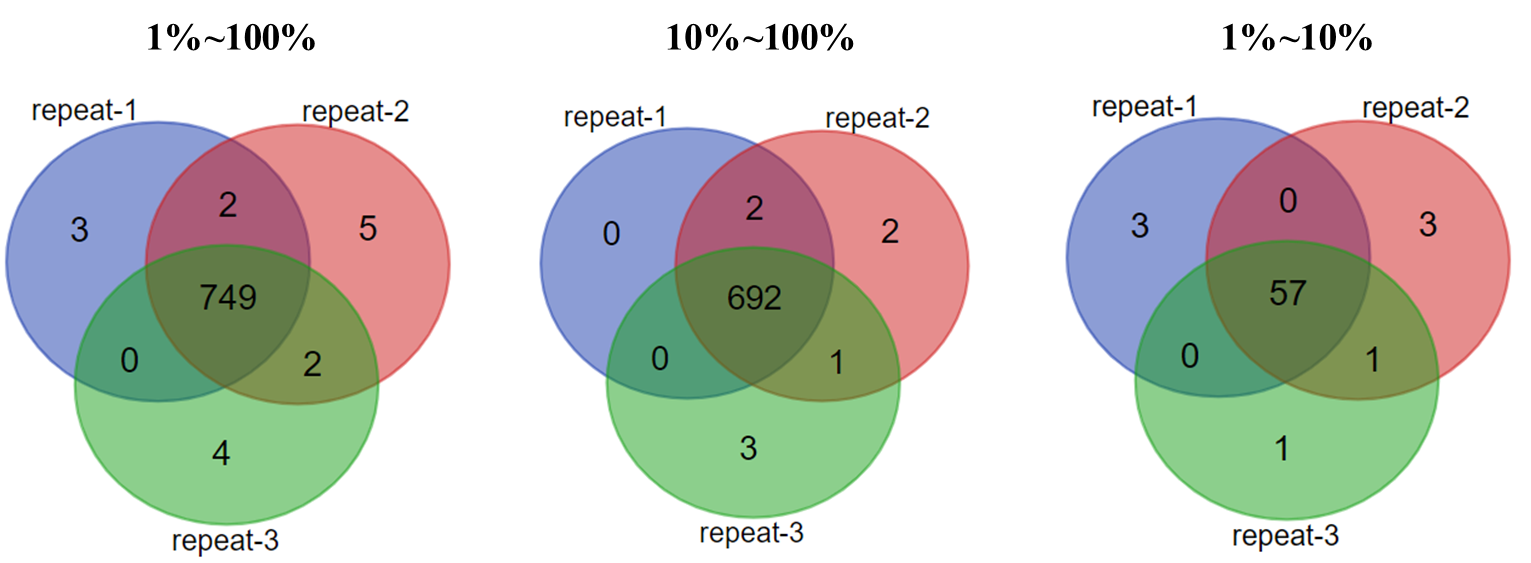


**Figure S1.** Venn diagram of mtDNA mutations with more than 1% heteroplasmy level in 24 fresh-frozen tissue samples by triple-repeated capture-based sequencing.


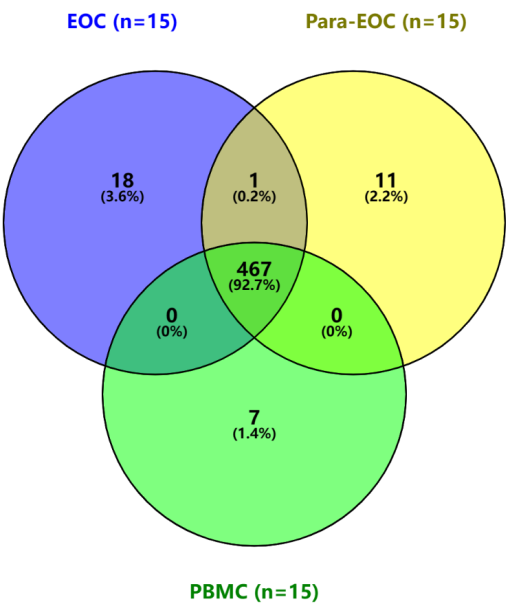


**Figure S2.** Venn diagram of mtDNA mutations with more than 1% heteroplasmy level in triple-paired tumor, para-tumor and PBMC samples from 15 EOC patients.


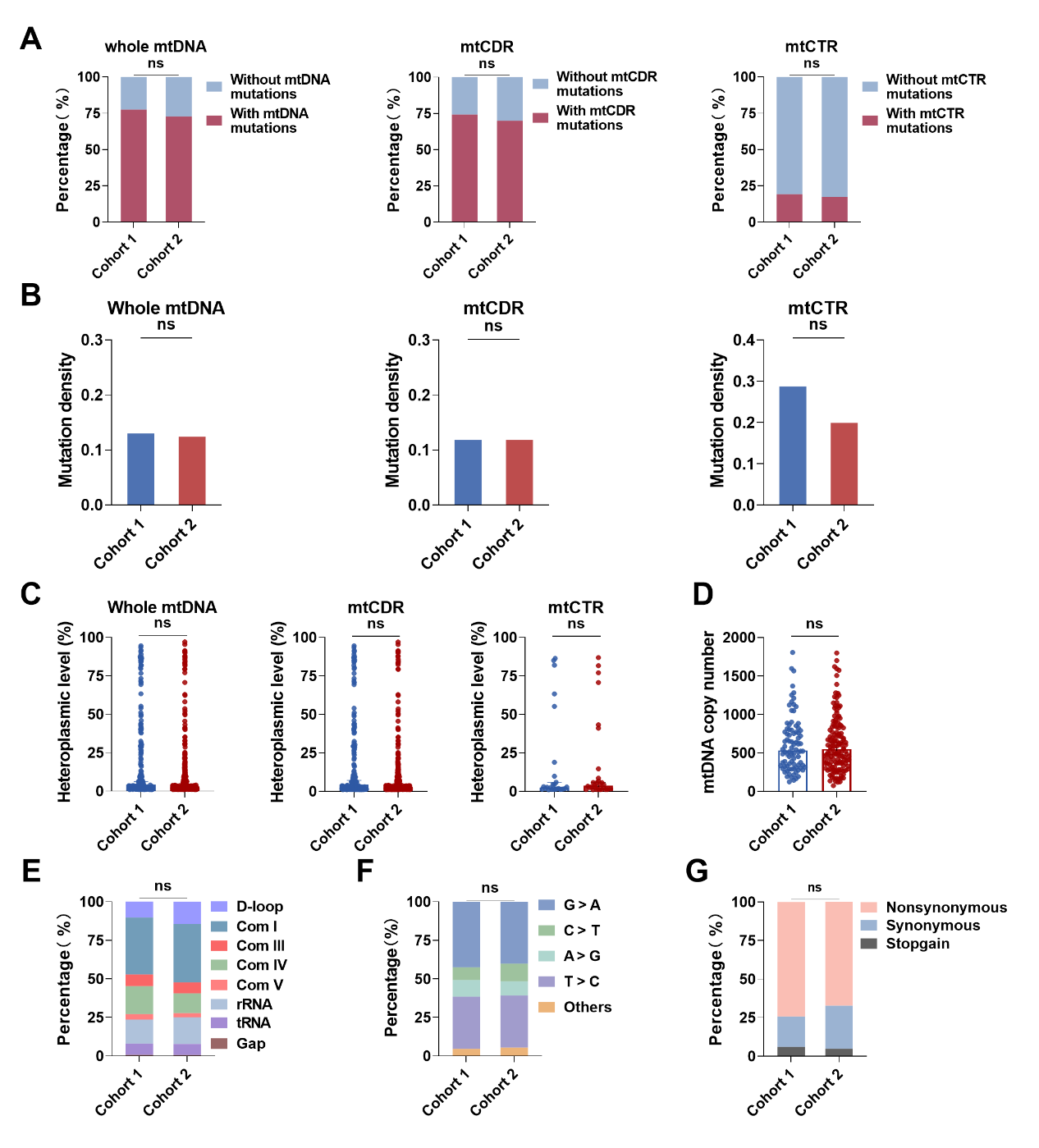


**Figure S3. The characteristics of mtDNA mutations were comparable between private EOC cohorts 1 and 2.**

(A) The percentage of EOC tissues with somatic mutations in mtDNA, mtCDR and mtCTR in private EOC cohorts.

(B-C) The mutation density and heteroplasmic level of somatic mutations in mtDNA, mtCDR and mtCTR in private EOC cohorts.

(D) The mtDNA copy number in EOC tissues from private EOC cohorts.

E. The percentage of somatic mtDNA mutations in the functional regions of mtDNA, including D-loop, respiratory complexes (Com I, Com III, Com IV, and Com V), rRNA, and tRNA genes.

(F) The percentage of base substitution types for somatic mtDNA mutations. Others denote all transversion mutations.

(G) The percentage of synonymous, nonsynonymous, and stopgain mtDNA mutations.

EOC, epithelial ovarian cancer; mtCTR, mtDNA control region; mtCDR, mtDNA coding region.

Data were expressed as mean ± SEM. Chi-square test was used for data analysis in (A-B) and (E-G). The Mann-Whitney U test was used for data analysis in (C-D).


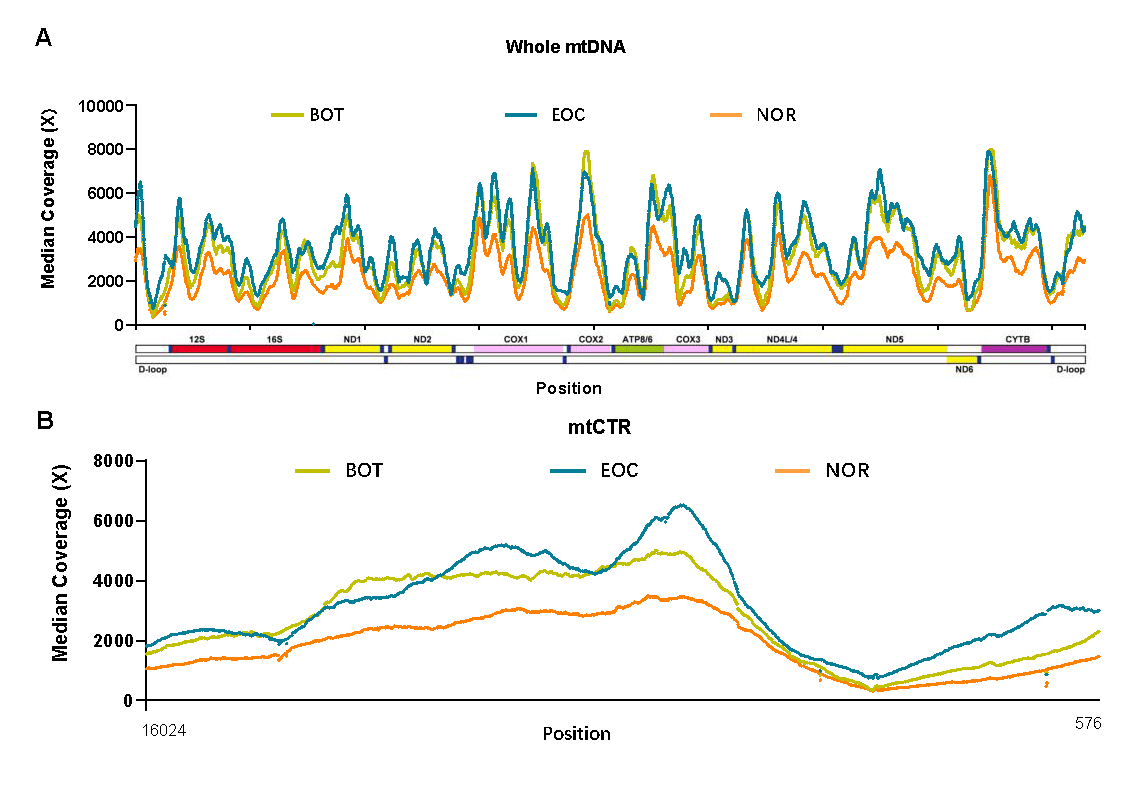


**Figure S4**. Median coverage of whole mtDNA (A) and mtCTR (B) in private EOC, BOT, and NOR cohorts. mtCTR, mtDNA control region.


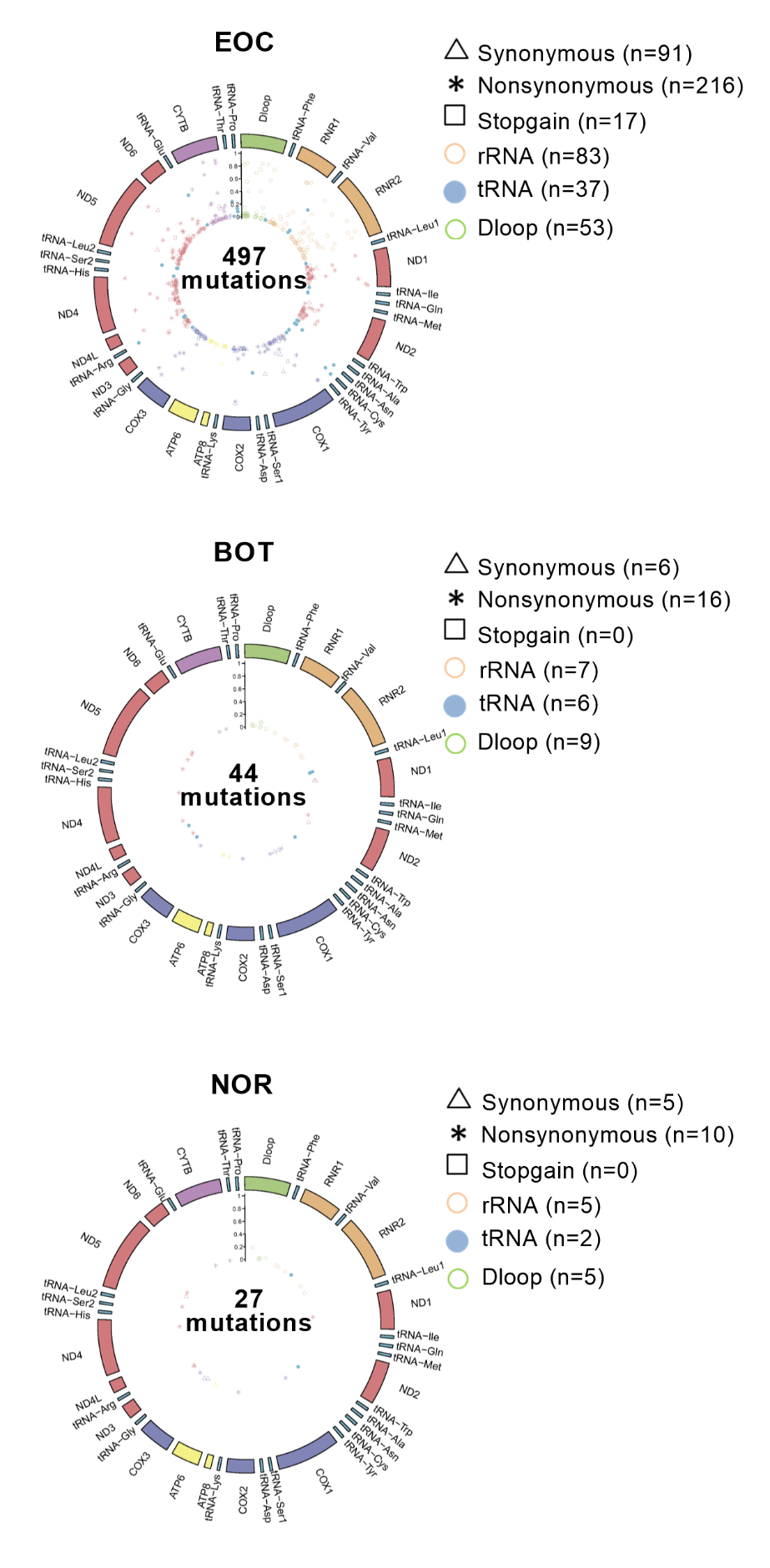


**Figure S5.** Circos plots showing the frequency and distribution of somatic mtDNA mutations from EOC (n = 239), BOT (n = 56), and NOR (n = 46) tissues in private cohort. Mutations distributed in the gap region were not shown.


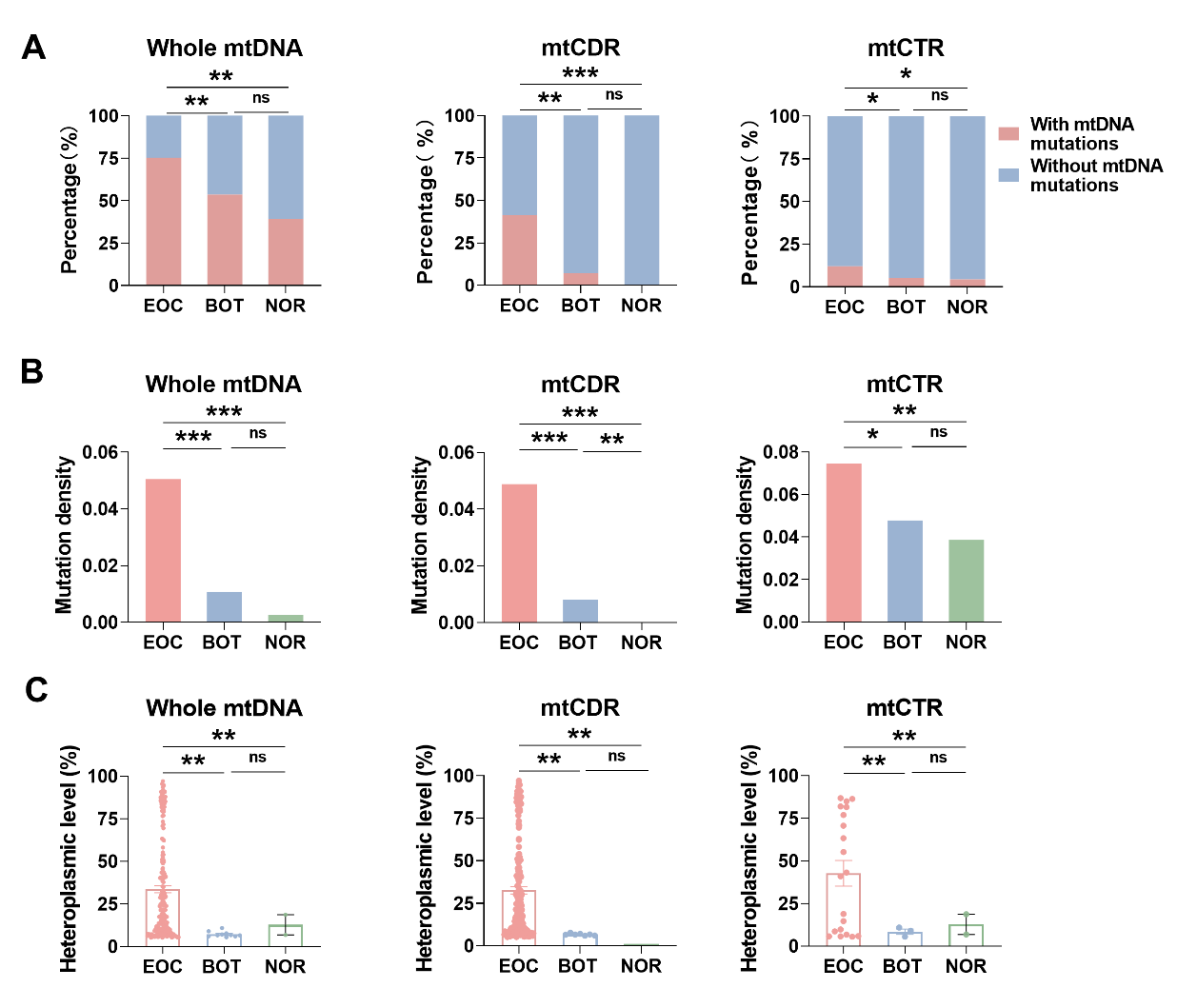


**Figure S6. Comparison of mtDNA mutations with VAF > 5% among different groups.**

(A) The percentage of tissues with somatic mutations (VAF > 5%) in mtDNA, mtCDR and mtCTR among three tissue types. (B-C) The mutation density and heteroplasmic level of somatic mutations (VAF > 5%) in mtDNA, mtCDR and mtCTR among three tissue types. Mutation density was calculated as the average number of mutations per sample per kilobase (kb). Data were expressed as mean ± SEM. Chi-square test was used for data analysis of the data in (A). One-way ANOVA with Bonferroni’s post hoc test was used for data analysis in (B-C). ^﹡^P < 0.05; ^﹡﹡^P < 0.01; ^﹡﹡﹡^P < 0.001.


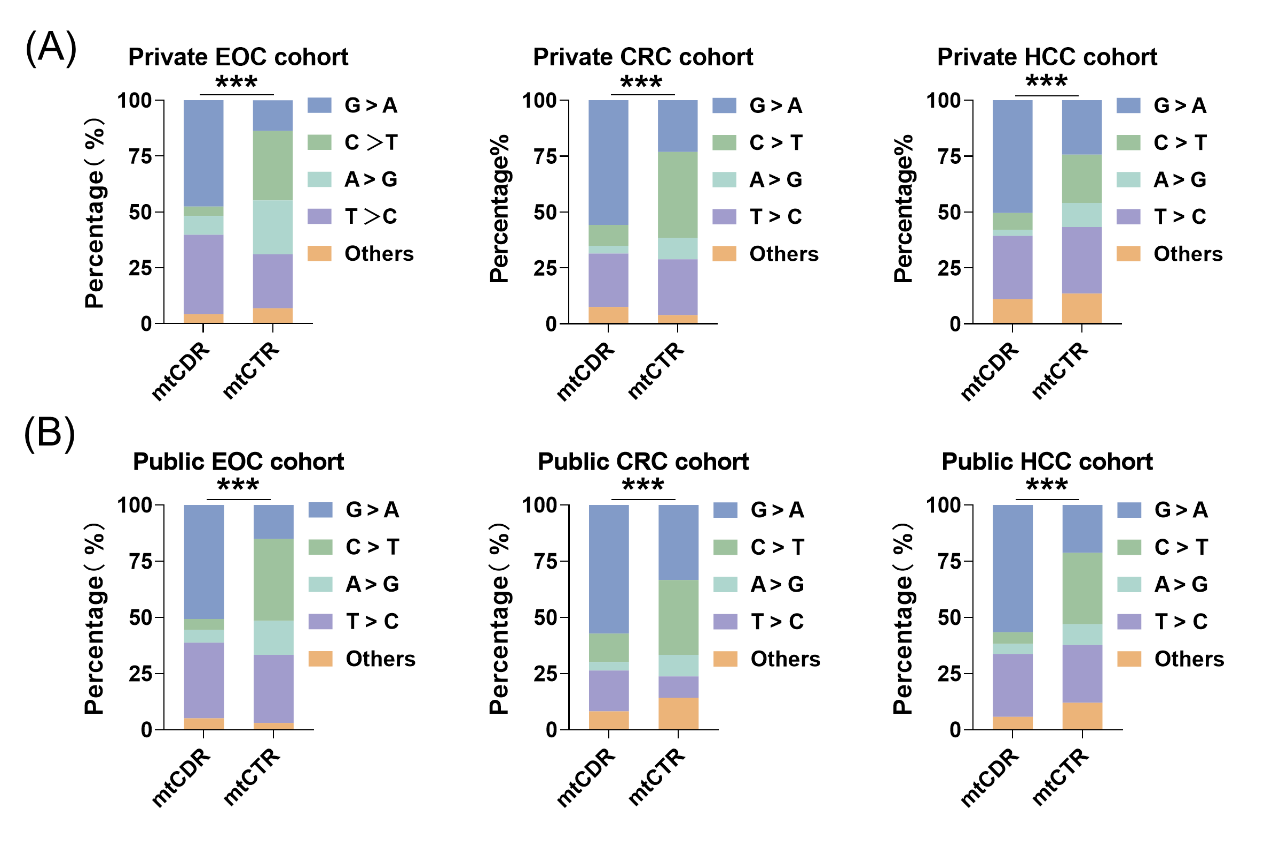


**Figure S7. MtDNA mutations in EOC showed strong region and strand biases.**

(A) Percentage of different substitution types in somatic mutations of mtCTR and mtCDR regions across three private cancer types.

(B) Proportion of different substitution types in somatic mutations of mtCTR and mtCDR regions across four public cancer types.

mtCDR, mtDNA coding region; mtCTR, mtDNA control region; ^﹡﹡﹡^*P* < 0.001.

Others denote all transversion mutations. Chi-square test was used for data analysis.


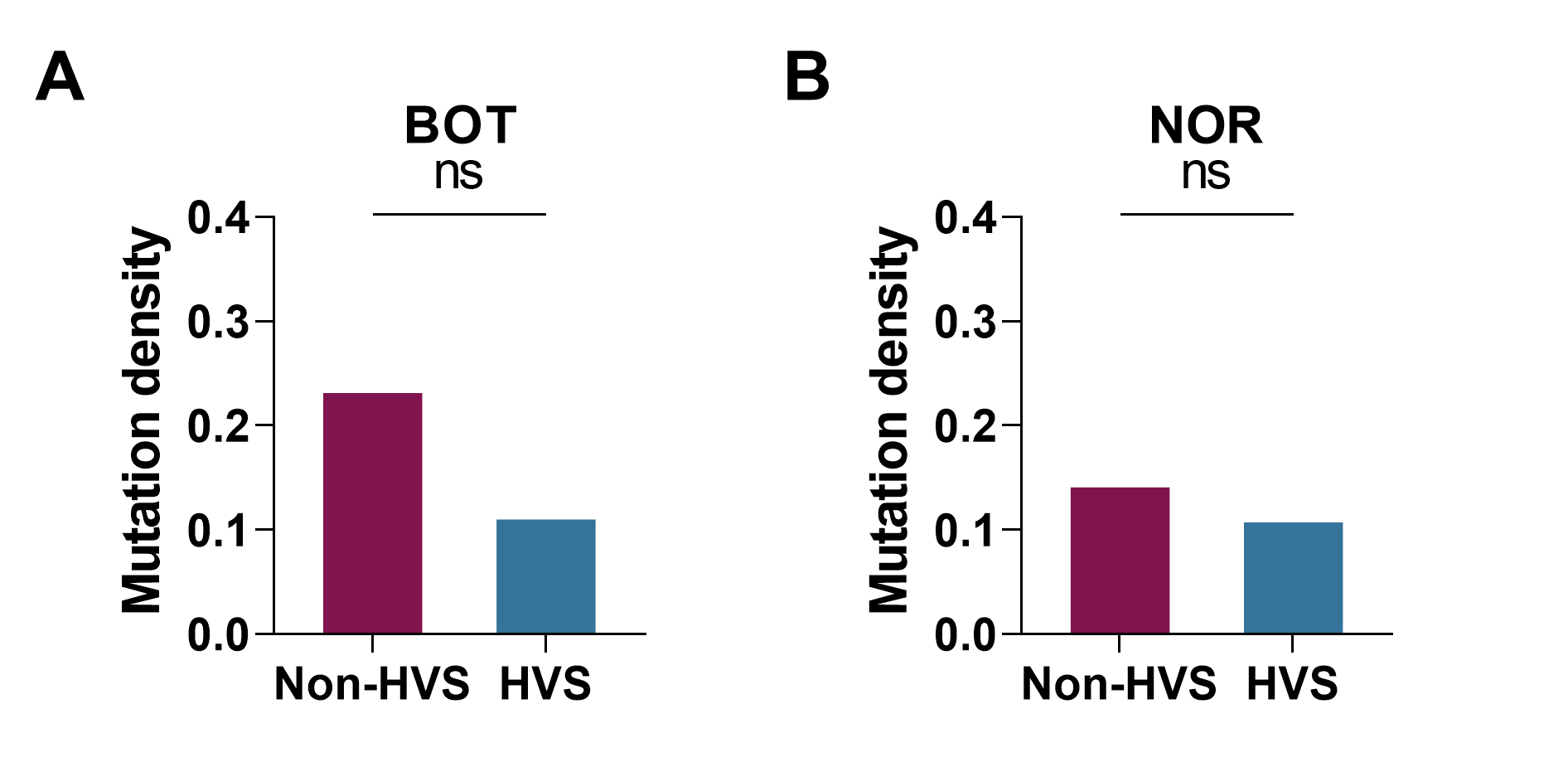


**Figure S8.** Mutation density of mtCTR non-HVS and HVS regions in private BOT and NOR cohorts. Chi-square test was used for data analysis.


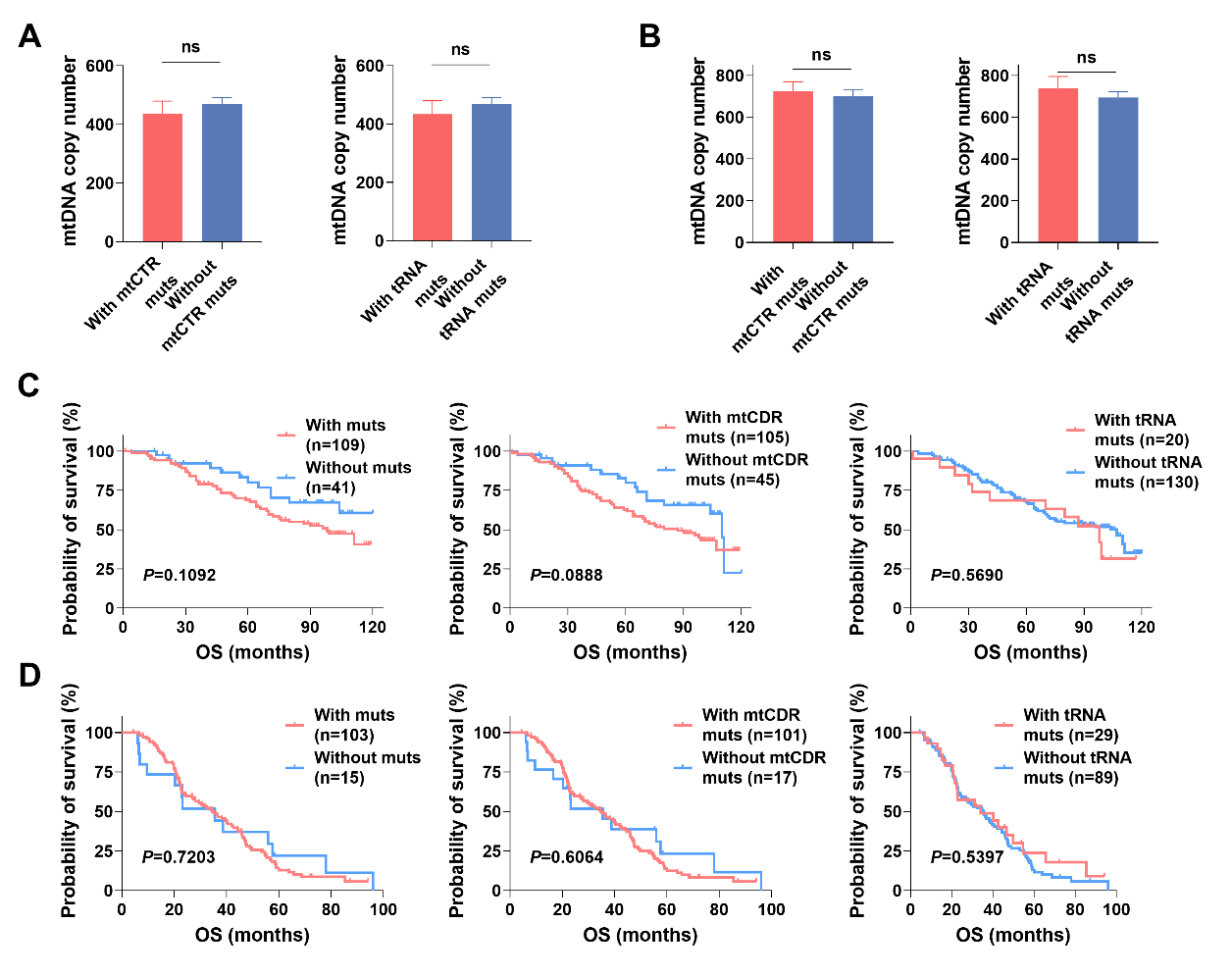


**Figure S9. The association between mtDNA mutations and mitochondrial biogenesis as well as clinical outcome of EOC**

(A-B) Comparison of mtDNA copy number between patients with and without mtCTR mutations, and patients with and without tRNA mutations in private (A) and public (B) EOC cohorts. (C-D) Kaplan-Meier curve analysis of overall survival (OS) between patients with and without mtDNA mutations, patients with and without mutations in mtCDR, and patients with and without tRNA mutations in private (C) and public (D) EOC cohorts.

**Table S1. Clinical characteristics of two private epithelial ovarian cancer (EOC), one private BOT, and one private NOR cohorts.**

| **Characteristics** | **Private**  **EOC**  **cohort 1 (n=89）** | **Private**  **EOC**  **cohort 2 (n=150）** | **Private BOT**  **cohort**  **(n=56)** | **Private NOR**  **cohort**  **(n=46)** | ***P*** |
| --- | --- | --- | --- | --- | --- |
| **Age, median (range)** | 55 (26-81) | 52 (21-83) | 51 (19-79) | 50 (18-80) | *P*=0.1878 |
| **FIGO stage, n (%)** |  |  | — | — | *P*=0.0876 |
| Ⅰ | 26 (29.2) | 53 (35.3) | — | — |  |
| Ⅱ | 5 (5.6) | 12 (8.0) | — | — |  |
| Ⅲ | 45 (50.6) | 77 (51.3) | — | — |  |
| Ⅳ | 13 (14.6) | 8 (5.4) | — | — |  |
| **CA125 status, n (%)** |  |  | — | — | *P*=0.5707 |
| <35 U/mL | 11 (12.4) | 24 (16.0) | — | — |  |
| ≥35 U/mL | 78 (87.6) | 126 (84.0) | — | — |  |
| **Histology, n (%)** |  |  | — | — | *P*=0.6856 |
| Serous | 66 (74.1) | 121 (74.7) | — | — |  |
| Mucinous | 13 (14.6) | 21 (14.0) | — | — |  |
| Clear cell | 3 (3.4) | 9 (6.0) | — | — |  |
| Endometrioid | 7 (7.9) | 8 (5.3) | — | — |  |

**Table S2. Summary of mtDNA sequencing data from private cohorts.**

| **Sample source** | **Sample type** | **No. of samples** | **Sequencing**  **platform** | **Sequencing**  **type** | **Average Q30** | **mtDNA** **coverage** | **Average depth (X)** |
| --- | --- | --- | --- | --- | --- | --- | --- |
| Private EOC cohort 1 | EOC | 89 | Illumina Novaseq | Capture-based | 95.2% | 100% | 5599±3069 |
|  | PBMC | 89 | Illumina Novaseq | Capture-based | 95.0% | 100% | 1494±1066 |
| Private EOC cohort 2 | EOC | 150 | Illumina Novaseq | Capture-based | 95.7% | 100% | 4912±3157 |
|  | Para-EOC | 150 | Illumina Novaseq | Capture-based | 95.4% | 100% | 4020±3186 |
| Private benign ovarian tumor cohort  (Private BOT cohort) | Benign ovarian tumor tissue | 56 | Illumina Novaseq | Capture-based | 94.4% | 100% | 4013±3054 |
|  | PBMC | 56 | Illumina Novaseq | Capture-based | 94.7% | 100% | 1478±987 |
| Private cohort without ovary tissue-associated disease | Normal ovary tissue | 46 | Illumina Novaseq | Capture-based | 93.9% | 100% | 3326±2789 |
|  | PBMC | 46 | Illumina Novaseq | Capture-based | 95.1% | 100% | 1576±1105 |
| Private CRC cohort | CRC | 432 | Illumina Hiseq | Capture-based | 93.5% | 100% | 5084±2357 |
|  | Para-CRC | 432 | Illumina Hiseq | Capture-based | 93.8% | 100% | 5200±2090 |
| Private HCC cohort | HCC | 110 | Illumina Hiseq | Capture-based | 93.2% | 100% | 9011±4575 |
|  | Para-HCC | 110 | Illumina Hiseq | Capture-based | 92.3% | 100% | 8656±4817 |

**Note:**

EOC: epithelial ovarian cancer tissue; Para-EOC: paired non-cancerous ovary tissue;

CRC: colorectal cancer tissue; Para-CRC: paired adjacent non-CRC colon/rectum tissue;

HCC: hepatocellular carcinoma tissue; Para-HCC: paired adjacent non-HCC liver tissue;

PBMC: peripheral blood mononuclear cell.

Private cohorts include two private epithelial ovarian cancers (EOC) cohorts, private ovarian benign tumor cohort, private cohort without ovary tissue-associated disease, private colorectal cancer (CRC) cohort, and private hepatocellular carcinoma (HCC) cohort.

| **Data source** | **VAF**  **cutoff** | **Sequencing**  **platform** | **Sequencing**  **type** | **Coverage**  **region** | **Heteroplasmy level data** | **Patient**  **type** | **No. of samples** | **mtCDR**  **Mutations**  **(No.)** | **mtCTR**  **Mutations**  **(No.)** |
| --- | --- | --- | --- | --- | --- | --- | --- | --- | --- |
| Nature  genetics  2020  (TCMA, Yuan et al) | ≥1% | Illumina | WGS | Whole  mtDNA | Available | Ovarian cancer  (OC) | 118 | 367 | 35 |
|  |  |  |  |  |  | Colorectal ccncer  (CRC) | 62 | 182 | 42 |
|  |  |  |  |  |  | Hepatocellular  carcinoma  (HCC) | 313 | 1045 | 170 |
|  |  |  |  |  |  | Breast cancer  (BC) | 217 | 589 | 99 |
| **Total** | | | | | | | **710** | **2183** | **346** |

**Table S3.** **Summary of mtDNA somatic mutations in public cohorts.**

**Note:** mtCDR, mtDNA coding region; mtCTR, mtDNA control region.
